# Boosting Antitumor Drug Efficacy with Chemically Engineered Multidomain Proteins

**DOI:** 10.1002/advs.201701036

**Published:** 2018-06-14

**Authors:** Seah Ling Kuan, Stephan Fischer, Susanne Hafner, Tao Wang, Tatiana Syrovets, Weina Liu, Yu Tokura, David Yuen Wah Ng, Andreas Riegger, Christina Förtsch, Daniela Jäger, Thomas F. E. Barth, Thomas Simmet, Holger Barth, Tanja Weil

**Affiliations:** ^1^ Max‐Planck Institute for Polymer Research Ackermannweg 10 55128 Mainz Germany; ^2^ Institute of Inorganic Chemistry I Ulm University Albert‐Einstein‐Allee 11 89081 Ulm Germany; ^3^ Institute of Pharmacology and Toxicology University of Ulm Medical Center Albert‐Einstein‐Allee 11 89081 Ulm Germany; ^4^ Institute of Pharmacology of Natural Products and Clinical Pharmacology Ulm University Helmholtzstraße 20 89081 Ulm Germany; ^5^ School of Materials Science and Engineering Southwest Jiaotong University 610031 Chengdu P. R. China; ^6^ Institute of Pathology Ulm University Albert‐Einstein‐Allee 23 89070 Ulm Germany

**Keywords:** chemically engineered proteins, combination oncotherapy, supramolecular fusion proteins, targeted delivery

## Abstract

A facile chemical approach integrating supramolecular chemistry, site‐selective protein chemistry, and molecular biology is described to engineer synthetic multidomain protein therapeutics that sensitize cancer cells selectively to significantly enhance antitumor efficacy of existing chemotherapeutics. The desired bioactive entities are assembled via supramolecular interactions at the nanoscale into structurally ordered multiprotein complexes comprising a) multiple copies of the chemically modified cyclic peptide hormone somatostatin for selective targeting and internalization into human A549 lung cancer cells expressing SST‐2 receptors and b) a new cysteine mutant of the C3bot1 (C3) enzyme from *Clostridium botulinum*, a Rho protein inhibitor that affects and influences intracellular Rho‐mediated processes like endothelial cell migration and blood vessel formation. The multidomain protein complex, SST3‐Avi‐C3, retargets C3 enzyme into non‐small cell lung A549 cancer cells and exhibits exceptional tumor inhibition at a concentration ≈100‐fold lower than the clinically approved antibody bevacizumab (Avastin) in vivo. Notably, SST3‐Avi‐C3 increases tumor sensitivity to a conventional chemotherapeutic (doxorubicin) in vivo. These findings show that the integrated approach holds vast promise to expand the current repertoire of multidomain protein complexes and can pave the way to important new developments in the area of targeted and combination cancer therapy.

## 1 Introduction

There is a pressing need to develop clinically effective chemotherapeutic treatment regimens that combine several drug molecules to exert their bioactivities by different mechanisms to overcome chemoresistance in oncotherapy.^1^, ^2^ Therapeutic antibodies and toxin enzymes are eminent candidates for multimodal treatment due to their highly specific modes of actions and thus noninterference with standard chemotherapeutics when used in combination.^3^, ^4^, ^5^ For instance, the clinically approved antiangiogenic monoclonal antibody bevacizumab (Avastin; registered trademark), which targets the vascular endothelial growth factor, has also been approved in combination with chemotherapeutic drugs^6^ such as doxorubicin (DOX) as first‐ and second‐line treatments of various cancer entities to improve their therapeutic efficacy.^7^, ^8^, ^9^ Nevertheless, side effects of bevacizumab such as kidney damage could arise^10^ and resistance inevitably develops;^11^, ^12^ thus, other targets, such as Rho GTPases that regulate alternative hallmarks of cancer, including cell migration, tumor suppression, and angiogenesis,^13^, ^14^ are also being considered as future strategies.^13^, ^15^, ^16^

**Figure 1:**
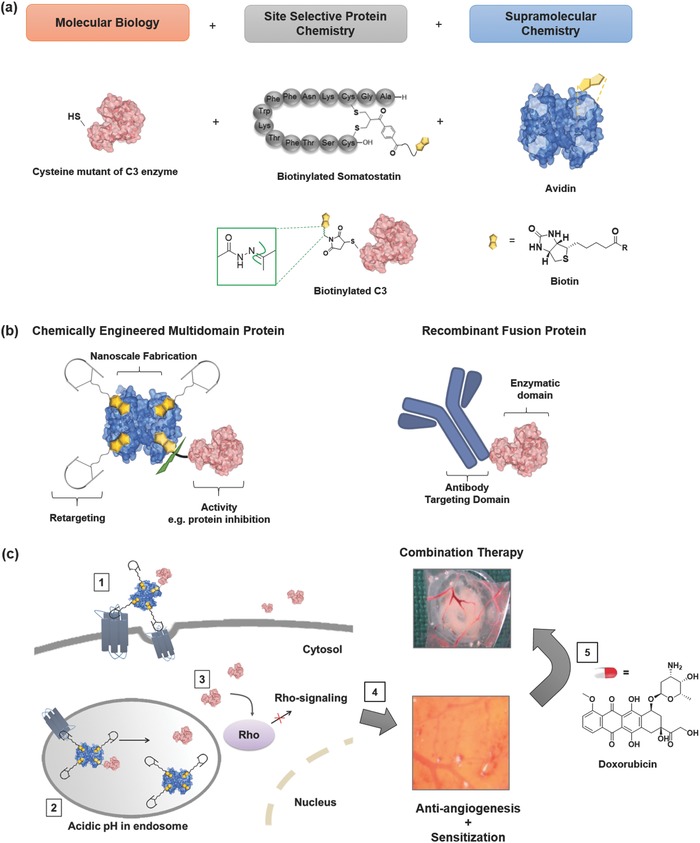
Chemically engineered multidomain protein, SST3‐Avi‐C3, and illustration of the bioactivity of SST3‐Avi‐C3 in tumor inhibition and application in combination with DOX to induce efficient cell death. a) The ensemble of site‐selective chemistry, supramolecular chemistry, and molecular biology is applied to prepare a synthetic multidomain protein complex with desirable features: avidin (blue) is used as the adaptor molecule for chemical fusion of the targeting entity, site‐selectively biotinylated (yellow) somatostatin (SST) to the potent Rho inhibitor (cysteine mutant of the C3 toxin, pink). A hydrazone linkage is introduced site selectively in the design for cleavage at acidic pH (structure in the green inset) to trigger C3 release inside cells. b) A recombinant multidomain antibody enzyme is shown as a comparison of their structural and functional features to the synthetic multidomain protein complex prepared in this study. c) A pictorial representation of the mode of action and synergistic effects of the combination of the chemical fusion toxin and the chemotherapeutic doxorubicin (DOX) in cancer cells: (1) internalization of SST3‐Avi‐C3 into cells via SSTR2‐mediated uptake, (2) cleavage of C3 in the acidic environment of endosomes, (3) release of C3 into the cytosol resulting in Rho inactivation leading to (4) reduced angiogenesis in tumor blood vessels and sensitization of the tumor cells for (5) subsequent co‐administration of conventional anticancer drug, e.g., DOX, finally resulting in efficient cell death at low dosage.

In this context, recombinantly engineered multidomain fusion proteins where potent toxin enzymes are fused to antibodies or to peptides targeting membrane receptors overexpressed on tumor cells to enhance selective cell uptake and reduce nonspecific toxicities are prominent alternatives to antibody treatments (**Figure** **1**).^17^, ^18^, ^19^, ^20^ However, the development of effective recombinant chimeras is often laborious;^17^ bioactivities can be reduced;^21^ and there are limited opportunities through biosynthesis to further improve pharmacokinetic parameters and therapeutic efficacy such as the attachment of stabilizing polymers or fusion of multiple copies of biomolecules, particularly cyclic peptides.^22^, ^23^, ^24^, ^25^, ^26^ Even though synthetic approaches have managed to overcome some of these technological hurdles, the application of chemical strategies alone may be insufficient to prepare multidomain protein conjugates with structural precision and bioactivity.^27^, ^28^ Thus, the synergistic combination of chemical and recombinant approaches to customize biotherapeutics could be attractive.^29^, ^30^, ^31^ Through such ensembles of chemical and biological tools, it could be possible to engineer synthetic multidomain protein complexes with rapid optimization to enhance physiological responses for their applications in multimodal therapy.

Herein, we report an integrated approach to combine bioactive entities at the macromolecular level into structurally ordered multidomain protein complexes to mitigate selective targeting of a hallmark protein molecule in cancer diseases (Figure 1): (1) the monodisperse avidin (Avi) is capitalized upon as a supramolecular “glue” for the precise nanoscale assembly of multiple copies of targeting peptides for enhanced cellular uptake and protein enzymes to achieve the desired therapeutic effects; (2) site‐specific chemistry is applied for the modification of the native, cyclic peptide hormone somatostatin (SST) for cellular retargeting as well as selective transport and controlled release of (3) a new cysteine mutant of the C3 enzyme from *Clostridium botulinum* into human cancer cells expressing SST‐2 receptors (SSTR2), consequently inhibiting tumor growth. SSTR2 are overexpressed by many solid tumor cell lines including lung cancer A549 cells and breast cancer MCF‐7 cells,^32^, ^33^, ^34^ and also by peritumoral veins of many solid tumors such as non‐small cell lung cancer (NSCLC).^35^ Thus, SST and its analogs have been applied as targets for both diagnostic and therapeutic purposes.^36^, ^37^, ^38^, ^39^ On the other hand, the C3 enzyme represents the only selective Rho‐A, ‐B, and ‐C inhibitors with high relevance for a broad range of cancer diseases and multimodal therapeutic options.^39^, ^40^, ^41^ Notably, the C3 protein (Cethrin; registered trademark) is already applied in therapy for treatment of spinal cord injuries, but its broader applications have been limited as it is not uptaken into most cell types.^42^ For toxin‐derived drugs, it was reported that although neutralizing antibodies can be formed in patients, treatment is still effective as reported for denileukin diftitox in treatment of cutaneous lymphoma^43^ and CRM197‐conjugates,^44^ and is unlikely to hamper their further clinical applications.

The synthetic multiprotein complex SST3‐Avi‐C3 prepared in this fashion exhibits selective cell uptake, specific inhibition of Rho in cancer cells, pH‐induced release into the cytosol of tumor cells and thereby significantly boosting the antitumor potency of a marketed anticancer therapeutic (Figure 1). In particular, in vivo studies with SST3‐Avi‐C3 clearly demonstrate significantly improved tumor inhibition at much lower dosage compared to bevacizumab, a first‐line treatment for advanced and metastasized NSCLC,^45^, ^46^ where the efficacy of chemotherapeutic drug regimens, such as DOX are severely hampered by adverse drug reactions and the onset of resistance.^47^ In addition, SST3‐Avi‐C3 co‐administration improves the effectiveness of DOX in A549 cells in vitro and NSCLC xenografts in vivo, underlining the therapeutic potential of the chemically engineered protein complex in the burgeoning field of combination therapy.

## 2 Results and Discussion

### 2.1 Preparation and Characterization of Multivalent SST(N)‐Avi (*N* = 1–4) Transporters

**Figure 2:**
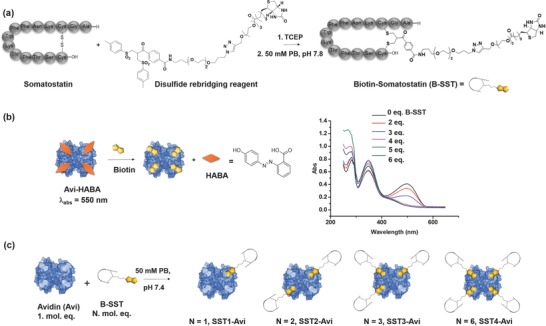
Synthesis of B‐SST by site‐selective disulfide modification and optimization to prepare SST(N)‐Avi conjugates. a) Synthesis of B‐SST by site‐selective disulfide rebridging: PB—phosphate buffer, TCEP—tris(2‐carboxyethyl)phosphine. b) Preformed Avi–HABA complex is displaced by biotin due to competitive binding and displacement can be observed by the change in absorbance at λ = 500 nm. The binding of B‐SST to Avi was monitored by HABA (inset) assay to determine the optimal stoichiometry for assembly of SST(N)‐Avi (*N* = 1–4): One mol. eq. of B‐SST is required per binding pocket in Avi. c) Preparation of SST(N)‐Avi from the reaction of Avi and B‐SST: 1–6 mol. eq. of B‐SST was added to a solution containing 1 mol. eq. of Avi in 20 × 10^−3^ m phosphate buffer, pH 7 to obtain the respective SST(N)–Avi complexes.

Avidin (Avi) is a tetrameric protein (pI > 9)^48^ that forms strong noncovalent interactions (*K*_d_ = 10^−15^ m) with its natural binding partner biotin in four binding sites^49^ and has been selected as a monodisperse supramolecular “glue” to combine different entities at the nanoscale. Additionally, although naturally occurring human antiavidin antibodies can be present in human serum, it has been established with oncology patients that this does not hamper the safety and efficacy, rendering it suitable for therapeutic application.^50^ As a first step, the transporter with the optimal number of the targeting peptide, SST, for efficient cellular transportation of enzyme cargo was determined. In order to assemble SST, site‐selective incorporation of biotin into SST is necessary. Disulfide rebridging represents an attractive chemical strategy that has been applied to selectively modify disulfide bonds in therapeutically native peptides and proteins with preservation of structural integrity and bioactivity.^51^, ^52^ Thus, SST which contains a single disulfide bridge was biotinylated in a site‐selective fashion using this contemporary bioconjugation strategy (**Figure** **2**a).^53^ In order to avoid a broad distribution of products, the optimal stoichiometry of somatostatin with terminal biotin (B‐SST) bound to Avi was characterized by displacing 2‐(4‐hydroxyphenylazo)benzoic acid (HABA)^54^ from the biotin‐binding sites. HABA has lower affinity for Avi than biotin and produces a characteristic absorbance at 500 nm in the complexed form (Figure 2c). The absence of the absorption peak when four equivalents of B‐SST had been added to Avi implies that each accessible biotin‐binding site was occupied with one equivalent of B‐SST.

**Figure 3:**
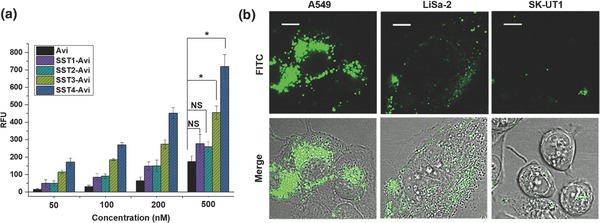
Multivalency effects observed for SST(N)–Avi complexes and LSCM analysis demonstrates the selective uptake of SST3‐Avi‐transporter into SSTR2‐positive human tumor cell lines. a) Cell uptake studies with A549 cells showing enhanced cellular uptake with increasing number of SST bound to Avi (*n* = 4, values are given as mean ± SD). The cells were incubated at 37 °C with each construct or with fluorescent Avi alone and the fluorescence was measured. Data were analyzed by one‐way analysis of variance (ANOVA) with Bonferroni correction for multigroup comparison at *p < 0.05, NS: not significant. Only statistical data for the concentration of transporters at 500 × 10^−9^ m are shown, full analysis for all concentrations is available in the Supporting Information (Figure S1, Supporting Information), RFU: relative fluorescence unit. b) A549, LiSa‐2 (both SSTR2‐positive), and SK‐UT1 (SSTR2‐negative) cells were incubated at 37 °C with SST3‐Avi (400 × 10^−9^ m). After 24 h, the uptake of FITC‐labeled SST3‐Avi was analyzed by immunofluorescence microscopy. Scale bars correspond to 10 µm.

Based on the optimization, the transporters, SST1‐Avi, SST2‐Avi, SST3‐Avi, and SST4‐Avi, with one to four B‐SST per Avi, respectively, were prepared by mixing the corresponding mole equivalents of B‐SST to fluorescently labeled Avi (Figure 2b). To determine the effect of the number of B‐SST on internalization, their uptake by human A549 lung carcinoma cells was investigated. A549 lung cancer cells were chosen for this study as they express the KRAS mutant of the Ras protein that deregulates RhoA signaling^55^ leading to cell transformation and increased resistance to chemical and biological therapies.^56^ A concentration dependency was observed for the SST(N)‐Avi transporters, with SST3‐Avi and SST4‐Avi exhibiting significant increase in cellular uptake compared to Avi (**Figure** **3**a; Figure S1, Supporting Information). The internalization into A549 cells was validated with laser scanning confocal microscopy (LSCM) (Figure S2, Supporting Information). Notably, there was a considerable increase in uptake when cells were incubated with SST3‐Avi or SST4‐Avi across all concentrations compared to SST1‐Avi and SST2‐Avi, in the order SST4‐Avi > SST3‐Avi > SST2‐Avi ≈ SST1‐Avi, suggesting a multivalency effect by which multiple ligands accomplish stronger target affinities compared to a single ligand.^57^, ^58^, ^59^ Thus, SST3‐Avi providing improved cellular uptake and a free available binding site for subsequent conjugation to the toxin enzyme was selected for further evaluation.

The selective internalization of SST3‐Avi into cells mediated by the SST receptor was confirmed using the SSTR2‐expressing human cancer cell line A549 and the SSTR2‐deficient cell line, SK‐UT1, and analyzed by LSCM. Besides the A549 cell line, an additional SSTR2‐positive human tumor cell line, LiSa‐2,^60^ was also evaluated as positive reference to substantiate SSTR2‐mediated uptake in receptor positive cell lines. The rapidly dividing LiSa‐2 cells are derived from a poorly differentiated, pleomorphic liposarcoma, a cancer that arises in fat cells in deep soft tissue. SST3‐Avi was efficiently taken up into A549 and LiSa‐2 cells, whereas no comparable uptake or binding of SST3‐Avi was observed in the receptor‐deficient SK‐UT1 cells, indicating that the uptake of SST3‐Avi proceeds by SSTR2 and exhibits selectivity toward SSTR2 overexpressing cells (Figure 3b; Figure S4, Supporting Information). The results are further corroborated by agonistic calcium flux assay (Figure S3, Supporting Information).

### 2.2 Synthesis of pH‐Cleavable Biotin–Maleimide Reagent (3) and Assembly of the Multiprotein Fusion Hybrid, SST3‐Avi‐C3

**Figure 4:**
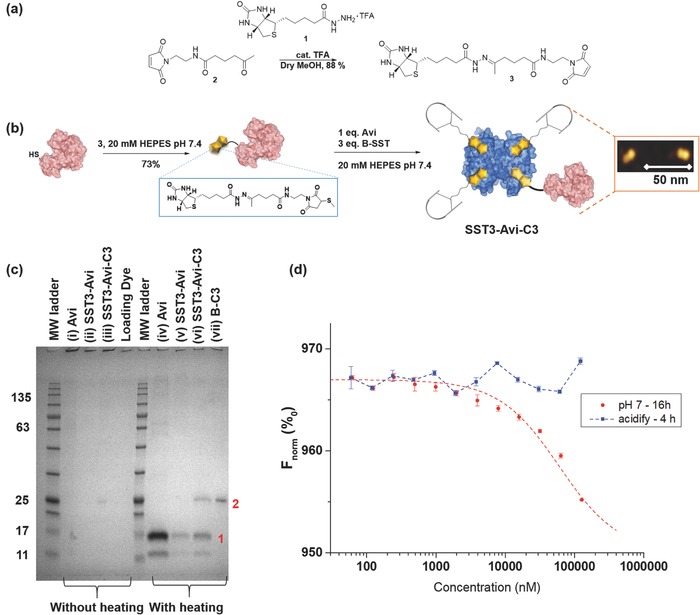
AFM and SDS‐PAGE analyses show the formation of the multidomain protein SST3‐Avi‐C3; SST3‐Avi‐C3 is cleaved at acidic pH. a) Synthesis of the biotin–maleimide reagent with hydrazone linkage (3). b) Biotinylation of C3 and preparation of the multidomain protein SST3‐Avi‐C3. Orange inset: AFM image showing formation of heterodimers. c) SDS‐PAGE analysis: Avi, SST3‐Avi, SST3‐Avi‐C3 and B‐C3 (25 kDa) without heating and with heating calibrated against a molecular weight marker (Applichem, Protein Marker VI). The proteins were stained and visualized using Imperial protein stain. Bands in lane are assigned as follow: 1) monomeric Avi (≈16 kDa) and 2) B‐C3 (≈25 kDa). d) Binding of B‐C3 to SST3‐Avi determined by microscale thermophoresis (*n* = 2, values are given as mean ± SD). A binding curve was observed even after 16 h incubation of fluorescent‐labeled B‐C3 and SST3‐Avi, indicating the stability of SST3‐Avi‐C3 (red). After acidification, no binding was observed, which suggests the release of the C3 (blue). The binding curve upon immediate incubation at pH 7 and binding to B‐C3 at pH 4 are available in the Supporting Information.

For the proof‐of‐concept, the C3 enzyme (pI > 9)^39^ from *C. botulinum*, an adenosine diphosphate (ADP)‐ribosyltransferase, and a Rho protein inhibitor was selected for conjugation.^42^ The toxin enzyme provides considerable therapeutic relevance in oncology to prevent endothelial cell migration and vessel formation in vitro and in vivo,^40^, ^61^ thereby regulating capillary formation and tumor angiogenesis. But its application has been significantly hampered by its very low uptake into most cell types,^42^, ^62^ and to date, only non cell‐type selective fusions of C3 enzyme have been reported.^62^, ^63^, ^64^ To chemically engineer the multiprotein complex, bacterial C3 enzyme has been attached to the SST3‐Avi by exploiting the high‐affinity supramolecular biotin–avidin interaction.^49^ Site‐selective mono‐biotinylation of C3 is an essential prerequisite to achieve the structurally defined multiprotein complex. Therefore, a new mutant of C3 was generated that harbors a cysteine residue at its N‐terminus. The resulting recombinant C3bot1‐A1C protein (henceforth C3) showed comparable enzymatic activity and Rho‐specific ADP‐ribosylation to wild‐type C3bot1, which can be used for the delivery approach (Figure S5, Supporting Information). Additionally, the introduction of a pH‐sensitive trigger that reacts to the acidic milieu in the endosomal microenvironment allows for subsequent intracellular release of the C3 cargo protein from the SST3‐Avi transporter molecule. The bifunctional maleimide–biotin conjugation reagent with a hydrazone linkage **3** was synthesized (**Figure** **4**a; Schemes S1 and S2, Supporting Information) and conjugated to C3 to obtain biotinylated C3 (B‐C3, Figure 4b). The controlled bioassembly of the multidomain SST3‐Avi‐C3 fusion construct was accomplished based on stoichiometric control of B‐C3, the protein Avi, and B‐SST according to the optimized ratio (B‐SST:Avi:B‐C3 = 3:1:1, Figure 4b). Complex formation was corroborated by analyses using sodium dodecyl sulfate polyacrylamide gel electrophoresis (SDS‐PAGE) and Western blot (Figure S7, Supporting Information), and further verified by atomic force microscopy (AFM) showing the formation of heterodimers (Figure 4b; Figure S6, Supporting Information). SDS‐PAGE analysis of SST3‐Avi‐C3 with and without heating (Figure 4c(iii,vi)), as reported previously,^26^, ^65^ against a known concentration of B‐C3 (Figure 4c(vii)) further reveals that nearly all free C3 have reacted during the conjugation reaction. The SDS‐PAGE results clearly excluded the electrostatic interaction between B‐C3 and Avi, and this is further supported by zeta potential measurements of B‐C3 and Avi, which showed comparable zeta values (Table S1, Supporting Information). The conjugation of SST3‐Avi to B‐C3 was further confirmed by microscale thermophoresis showing a binding curve upon immediate incubation and the construct remained bound even after 16 h, substantiating the stability of SST3‐Avi‐C3 in pH 7 buffer (Figure 4d; Figure S8, Supporting Information). Thereafter, the solutions were acidified to induce cleavage of SST3‐Avi‐C3 and there was no binding observed, thereby confirming the release of C3 in acidic media (Figure 4d). The structural integrity and stability of SST3‐Avi‐C3, up to 16 h, in human serum were investigated using Western blot analysis, and no increase in B‐C3 or biotinylated peptide fragments due to proteolytic cleavage was observed, indicating the stability of the multidomain protein (Figure S9, Supporting Information).

### 2.3 Selective Delivery of C3 into SST2 Receptor Expressing A549 Cancer Cells by the SST3‐Avi Transporter

**Figure 5:**
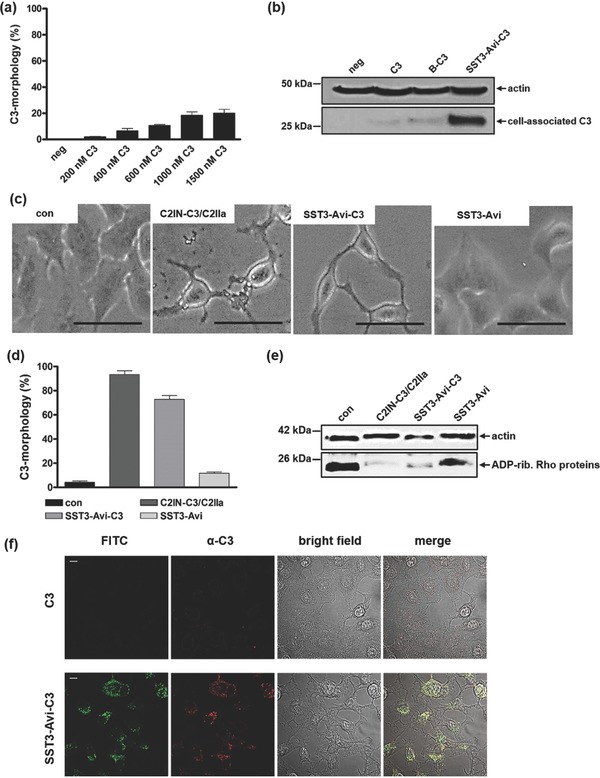
Treatment of A549 cells with SST3‐Avi‐C3 results in the efficient delivery of C3 accompanied by the characteristic C3‐induced changes in cell morphology due to the C3 catalyzed Rho–ADP‐ribosylation. a) A549 cells were incubated at 37 °C for 24 h with different concentrations of C3 to exclude morphological changes due to C3 alone. b) A549 cells were treated with C3, B‐C3, and SST3‐Avi‐C3 (350 × 10^−9^ m solutions) or were left untreated. After 24 h, cells were washed, lysed, and cell‐associated C3 was detected using a C3 antibody. Equal protein loading was verified by antiactin immunoblotting. c) A zoom‐in of phase contrast images show the C3‐induced change of the cell morphology when SST3‐Avi‐C3 and the positive control, C2IN‐C3/C2IIa, were applied. Scale bars correspond to 50 µm. d) Cultured A549 cells were treated with C2IN‐C3/C2IIa (100/160 × 10^−9^ m), SST3‐Avi (400 × 10^−9^ m), SST3‐Avi‐C3 (350 × 10^−9^ m), or were left untreated for quantification. For quantitative analysis, the percentage of A549 cells showing C3 morphology was determined from three independent samples treated in the same way (*n* = 3, values are given as mean ± SD). e) C3 coupled to SST3‐Avi is taken up efficiently into cells and leads to ADP‐ribosylation of Rho proteins in the cytosol of A549 cells. A549 cells were incubated with SST3‐Avi‐C3 (350 × 10^−9^ m) for 24 h at 37 °C. For control, cells were left untreated or were incubated with either SST3‐Avi (400 × 10^−9^ m) or C2IN‐C3/C2IIa (100/160 × 10^−9^ m). Cells were washed, lysed, and lysates were incubated with biotin‐labeled NAD^+^ (10 × 10^−6^ m) and fresh C3 (300 ng) for 30 min at 37 °C. Next, lysates were subjected to SDS‐PAGE and Western blot to investigate the amount of biotin‐labeled ADP‐ribosylated Rho, using peroxidase‐coupled streptavidin. Equal protein loading was again verified by antiactin immunoblotting. f) LSM analysis showing internalization of C3 mediated by SST3‐Avi. A549 cells were incubated at 37 °C with SST3‐Avi‐C3 (350 × 10^−9^ m). After 24 h, cells were washed, fixed, permeated, and stained with a primary rabbit‐antibody against C3 (α‐C3) and visualized using an Alexa 647‐coupled secondary antibody (red). The transporter was labeled with FITC (green). Scale bars correspond to 10 µm.

Having confirmed the identity and stability of the SST3‐Avi‐C3 hybrid, we investigated whether the SST3‐Avi transporter effectively delivers biologically active C3 enzyme into A549 lung cells to achieve Rho inactivation. A549 cells were incubated with increasing amounts of C3 to determine typical morphological changes due to the internalization of C3. Even at very high concentrations, C3 had only minimal effects on the morphology of A549 cells (**Figure** **5**a). Next, we incubated A549 cells with C3, B‐C3, and SST3‐Avi‐C3 and analyzed the cell‐associated C3 after SDS‐PAGE and Western blotting using a specific C3 antibody. The results show a strong C3 signal only when C3 is conjugated to the transporter platform indicating the necessity of the SST3‐Avi transporter for cellular uptake of C3 (Figure 5b). C3‐catalyzed Rho inactivation results in a reorganization of the actin cytoskeleton and a characteristic change in cell morphology, which is a well‐established endpoint to monitor the uptake of C3 into the cytosol.^66^, ^67^ Thus, A549 cells were incubated with SST3‐Avi‐C3 or with the SST3‐Avi transporter alone. As controls, cells were treated with the established recombinant cell‐permeable C3‐fusion toxin C2IN‐C3/C2IIa or left untreated.^68^ Noteworthy, C2IN‐C3/C2IIa is not cell‐type selective and is therefore taken up into various cells including non‐tumorous cells. As shown in Figure 5c,d, only cells treated with C2IN‐C3/C2IIa or with SST3‐Avi‐C3 showed the typical C3‐induced change of their morphology, whereas the transporter alone had no effect. Biochemical analysis shown in Figure 5e demonstrates that Rho was ADP‐ribosylated in cells, either treated with C2IN‐C3/C2IIa or SST3‐Avi‐C3, but not in cells treated with SST3‐Avi or C3 alone. In this assay, a weak signal in the Western blot indicates that most of the Rho protein was already ADP‐ribosylated in the living cells by the internalized C3 and is therefore no longer available for the subsequent in vitro ADP‐ribosylation reaction with biotinylated nicotinamide adenine dinucleotide (NAD) as co‐substrate.^68^ Both the cell morphology and biochemical analyses indicate that enzymatically active C3 was present in the cytosol of these cells, i.e., C3 was efficiently delivered into the host cell cytosol by the SST3‐Avi transporter. The SST‐mediated delivery and internalization of C3 enzyme into A549 cells and the resulting C3‐induced changes in cell morphology were also clearly detectable and confirmed by LSCM (Figure 5f; Figure S10, Supporting Information). However, the molecular mechanisms underlying the translocation of C3 proteins across endosomal membranes into the cytosol are not known and might be specific for monocytic cells as target cells for C3 proteins.

### 2.4 Antiangiogenic Activity of SST3‐Avi‐C3 in Non‐Small Cell A549 Lung Adenocarcinoma Xenotransplants

The intratumoral injection of anti‐Rho small interfering ribonucleic acid (siRNA) has previously been demonstrated to almost completely prevent proliferation and angiogenesis of cancer xenografts in a rodent model, rendering Rho‐targeting an eminent strategy for next‐generation oncotherapy.^15^ Thus, the impact of Rho inhibition by SST3‐Avi‐C3 on tumor‐associated angiogenesis in NSCLC tumors was studied in vivo, using the chick chorioallantoic membrane (CAM) for xenotransplantation of A549 NSCLC cells. This model has been previously reported and validated for the evaluation of anticancer agents and has been recommended by the United States Food and Drug Administration as a preclinical model for in vivo studies on angiogenesis.^69^, ^70^, ^71^ In addition, the CAM serves as a nutritious natural substrate for the cells' growth and allows the 3D formation of solid tumors in an in vivo microenvironment, as well as a reproducible and efficient model^72^, ^73^ conforming to the 3R principle to reduce mammalian experiments (see the Supporting Information for further description).

**Figure 6:**
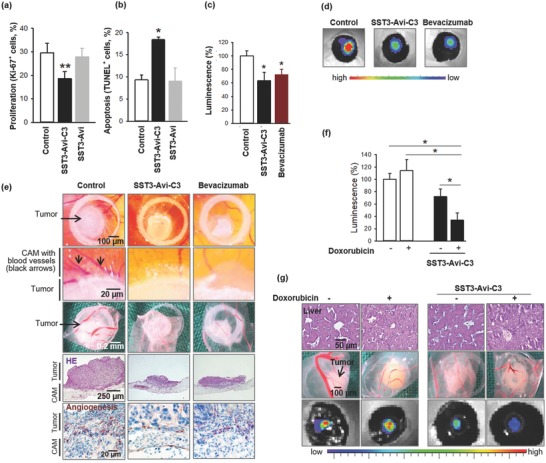
Selective growth inhibition of A549 lung cancer xenografts, antiangiogenic properties of SST3‐Avi‐C3, and enhanced antitumor efficacy of co‐administration of SST3‐Avi‐C3 with DOX in vivo. a,b) 1 × 10^6^ A549 cells were xenotransplanted on the chorioallantoic membrane of chick eggs 8 d after fertilization. One day later, 20 µL of SST3‐Avi‐C3 (350 × 10^−9^ m) and of controls (NaCl 0.9%, 400 × 10^−9^ m SST3‐Avi) were topically applied. After 24 h, tumor xenografts were collected and histopathologically analyzed. For calculation of the proportion of marker positive and negative cells, 200–790 cells per tumor were evaluated. Data are mean ± standard error of the mean (SEM) of 11–13 eggs per group. a) Ki‐67^+^ proliferating cells in A549 lung cancer xenografts. Newman–Keuls test, **p < 0.01. b) TdT^+^ apoptotic cells within tumor xenografts. Newman–Keuls test, *p < 0.05. c–e) To further evaluate the antiangiogenic properties of SST3‐Avi‐C3, 1 × 10^6^ tumor cells stably expressing firefly luciferase were xenografted onto the CAM and topically treated with 20 µL of either SST3‐Avi‐C3 (350 × 10^−9^ m), the antiangiogenic drug bevacizumab (34 × 10^−6^ m), or control (NaCl 0.9%). Bioluminescence was measured 24 h later, 15 min after topical addition of 10 µL d‐luciferin (0.75 mg mL^−1^) with an integration time of 1 s. c) Tumor growth was analyzed by expression of luciferase of cancer xenografts measured by an IVIS in vivo imaging system. The values are mean ± SEM of *n* = 4–6, 100% = 1.57 × 10^8^ photons s^−1^ cm^−2^ sr^−1^. Newman–Keuls test, *p < 0.05. d) Representative IVIS pictures of luciferase expressing tumor xenografts in ovo. e) Representative macroscopic pictures of whole cancer xenografts in ovo (upper row), enlarged at the transition zone between tumor and CAM (second row), tumors after removal (third row), histochemical sections of tumors (fourth row, HE, original magnification 50×) and angiogenesis marker desmin at the transition area of tumor and CAM (bottom row, original magnification 200×). f,g) For co‐administration studies, xenografts grown as described in panel (c) were treated with 20 µL of either SST3‐Avi‐C3 (350 nm) or NaCl 0.9% with or without DOX (100 × 10^−6^ m). Bioluminescence was measured 24 h later. f) Tumor growth was monitored by luciferase activity of cancer xenografts as measured by an IVIS in vivo imaging system. Data are mean ± SEM of *n* = 4–12 eggs per group, 100% = 1.35 × 10^9^ photons s^−1^ cm^−2^ sr^−1^. Newman–Keuls test, *p < 0.05. g) Representative sections of chick embryo liver (upper row) and pictures of removed A549 cancer xenografts (center row) and luciferase expressing lung cancer xenografts in ovo (bottom row).

The topical application of SST3‐Avi‐C3 onto the tumor‐bearing area of the CAM 24 h after xenotransplantation led to reduced NSCLC tumor growth as evidenced by a significant reduction in the number of NSCLC cells expressing the proliferation marker Ki‐67, a concomitant significant increase of tumor cells undergoing apoptosis terminal deoxynucleotidyl transferase dUTP nick end labeling (TUNEL) positive (**Figure** **6**a,b) and no effect on tumor growth in ovo from the single components of the transporter, SST3‐Avi or B‐C3, alone (Figure 6; Figures S11 and S12, Supporting Information). However, when analyzed in vitro after 24 h incubation (Figure S13, Supporting Information) SST3‐Avi‐C3 does not affect the viability of A549 cells, suggesting that these effects on tumor growth could be indirectly induced, e.g., by interaction with the tumor environment. Noteworthy, the histopathological analysis of chick embryo hepatic tissue 24 h after SST3‐Avi‐C3 application did not provide any indication of systemic toxicity (Figure 6g; Figure S11b, Supporting Information); likewise, there were no signs of macropathological damage in the chick embryos, as evidenced by histological examination of chick hepatic morphology. We further observed considerable reduction of peritumoral blood vessel formation in SST3‐Avi‐C3‐treated xenografts (Figure S11, Supporting Information). This effect only occurred when the intact multiprotein complex SST3‐Avi‐C3 was applied and it did not occur when the individual components, namely the SST3‐Avi transporter or B‐C3, were applied (Figures S11 and S12, Supporting Information). These results clearly indicate that SST3‐Avi‐C3‐mediated inhibition of neoangiogenesis depends on both, (1) the somatostatin to address SSTR‐expressing target cells and induce efficient cellular uptake and (2) the Rho‐modulating C3 toxin moiety to affect intracellular mechanisms.

The antiangiogenic efficacy of SST3‐Avi‐C3 to counteract tumor growth was directly compared with the clinically approved antibody bevacizumab. SST‐Avi‐C3 and bevacizumab were applied on pre‐established NSCLC xenografts stably transfected with firefly luciferase. After 24 h treatment, xenograft bioluminescence as an indicator of tumor cell growth was significantly reduced in the SST3‐Avi‐C3‐treated group already at very low concentration (350 × 10^−9^ m). In the positive control group, the antibody bevacizumab induced comparable effects only at much higher concentration, i.e., at 34 × 10^−6^ m (about 100 times higher, Figure 6c,d). In tissue sections, we visualized the microvessel maturation within tumor xenografts by immuno‐histochemical staining of desmin. Desmin‐positive pericytes are associated with high‐grade cancer disease and are commonly used as a biomarker of angiogenesis^74^ in the CAM assay.^75^ Remarkable antiangiogenic efficacy of SST3‐Avi‐C3 (Figure 6e) was confirmed by only marginal expression of desmin in SST3‐Avi‐C3‐ and bevacizumab‐treated tumors compared to the untreated controls after staining of desmin‐positive pericytes in tumor xenografts. The specific inhibition of Rho by C3 toxin successfully repressed tumor vascularization leading to this pronounced effect.

### 2.5 Increased Efficacy of Doxorubicin after Pretreatment of Non‐Small Cell A549 Lung Adenocarcinoma Xenotransplants with SST3‐Avi‐C3

Besides the regulation of cell migration, angiogenesis and tumor suppression, Rho inhibition is emerging as a strategic approach for the sensitization of cancer cells to enhance the anticancer efficacy of existing chemotherapeutics toward tumors with limited treatment options such as NSCLC^76^, ^77^ and is of high clinical relevance.^78^, ^79^ Having shown that SST3‐Avi‐C3 is much more potent compared to bevacizumab against A549 lung cancer cells with about 100 times reduction in required dosage, co‐administration of SST3‐Avi‐C3 with the drug DOX, a first‐line chemotherapeutics in oncotherapy, was investigated. Preincubation of A549 cells with SST3‐Avi‐C3 followed by application of DOX considerably reduced cell viability (Figure S14, Supporting Information) compared to DOX treatment alone. By contrast, no enhancement of the DOX efficacy was observed after pretreatment with the transporter, SST3‐Avi. These results imply that lower DOX doses can be applied through specific C3‐mediated Rho inhibition to achieve similar DOX cytotoxicity and offers a valuable strategy for the sensitization of cancer cells through a different intracellular targeting mechanism compared to marketed therapeutics such as bevacizumab. Co‐administration of antiangiogenic SST3‐Avi‐C3 and DOX was tested in vivo in the CAM xenotransplantation model, and significantly reduced bioluminescence of luciferase‐transfected lung cancer xenografts was observed when compared to either monotherapy (Figure 6f,g).

## 3 Conclusion

In summary, we report the chemical engineering of a customized multidomain protein complex SST3‐Avi‐C3 through controlled nanoassembly that displays rich bioactivities. Each protein domain contributed unique bioactivity features to the resulting construct: (1) SST provides cell‐type selectivity for cells overexpressing SSTR2; (2) multiplication of the SST targeting groups enhanced cellular uptake via the multivalency effect; and (3) potent enzyme activity of the only known selective Rho inhibitor, bacterial toxin C3, that is (4) released upon a pH stimulus in acidic cellular compartments to significantly affect tumor angiogenesis by preventing blood vessel formation and regulation of capillary formation.

The chemically engineered transporter domain SST3‐Avi was structurally optimized for enhanced cellular uptake into SSTR2 positive cells. SST3‐Avi provides a single free biotin binding site for attaching biotinylated C3, which was transported selectively into two distinctively different cancer cells, A549 as well as LiSa‐2 cells, by receptor‐mediated uptake. Controlled biomolecular assembly of multiple SST and C3 with molecular precision yielded the potent chemically engineered multidomain protein SST3‐Avi‐C3 that affords its high anticancer efficacy in a preclinical model of treatment‐resistant non‐small cell lung carcinoma xenografts. In comparison to the clinically approved antiangiogenic drug bevacizumab, SST3‐Avi‐C3 exerts improved bioactivity by an alternative pathway inhibiting Rho‐dependent tumor‐associated angiogenesis at a much lower therapeutic dosage. When applied in concomitant treatment with the existing chemotherapeutic DOX, SST3‐Avi‐C3 significantly reduced tumor growth of cultured human tumor cells and A549 xenografts in vivo.

With the burgeoning interest to increase the efficacy of prevailing chemotherapeutics through addressing intracellular molecular targets to sensitize cancer cells in multimodal treatments, our findings hold tremendous potential to harness therapeutically attractive protein enzymes such as the C3 enzyme for tumor growth inhibition and minimum drug dosage applied in combination with marketed chemotherapeutics. Moreover, the integrated chemical platform presented herein can be customized to interface a variety of bioactive entities with therapeutically attractive enzymes to produce unique chemically engineered multidomain proteins that could not be achieved by chemical or recombinant technologies alone. We envision immense potential for rapid optimization to expand the repertoire of therapeutic multidomain protein complexes thus opening new avenues for the innovation of next‐generation high‐efficacy oncological treatment.

## 4 Experimental Section

General information and methods are provided in the Supporting Information.

*Optimization of Biotinylated Somatostatin (B‐SST)‐Avidin Binding*: Native avidin (Novabiochem) and B‐SST^53^ (0–6 eq.) were dissolved in phosphate buffer (50 × 10^−3^ m, pH 7.4) to afford 1 mg mL^−1^ avidin solutions with different equivalents of B‐SST. The respective solutions (100 µL) and HABA solution (1 mg mL^−1^ in dimethyl sulfoxide, 2 µL) were introduced into a UV‐star flat bottom 384‐well plate (Greiner bio‐one, Frickenhausen, Germany). The mixture was mixed and the absorbance spectrum (250–650 nm) was measured.

*Preparation of Transport Proteins SST1‐Avi, SST2‐Avi, SST3‐Avi, SST4‐Avi*: Avidin (10 mg) was dissolved in 10 mL of phosphate buffer (50 × 10^−3^ m, pH 7.4) and rhodamine B isothiocyanate (1 mg mL^−1^ in *N*,*N*‐dimethylformamide (DMF), 406 µL) was added. The reaction mixture was stirred overnight and dried via lyophilization. The mixture was purified by size exclusion chromatography using Sephadex G‐25 matrix to afford 10 mg of rhodamine‐labeled avidin (Rh‐Avi) with quantitative yield. Rh‐Avi (2 mg) and different equivalents of B‐SST (1–3 eq. and 6 eq.) were dissolved in phosphate buffer (50 × 10^−3^ m, pH 7.4) separately, and the reaction mixtures were incubated for 1 h. The mixtures were purified by size exclusion chromatography using Sephadex G‐25 matrix to afford SST1‐Avi, SST2‐Avi, SST3‐Avi, SST4‐Avi with quantitative yields.

*Cell Culture*: A549, SK‐UT‐1 (DSMZ, German Collection of Microorganisms and Cell Cultures), and LiSa‐2^60^ cells were cultivated at 37 °C and 5% CO_2_ in Dulbecco's modified Eagle medium (DMEM, high glucose). Media contained 10% heat inactivated fetal calf serum (FCS), l‐glutamate (2 × 10^−3^ m), and 100 U mL^−1^ penicillin and 100 µg mL^−1^ streptomycin. Cells were routinely trypsinized and reseeded twice per week. For the experiments, cells were seeded in plastic dishes and incubated with the respective compounds in the medium at 37 °C and 5% CO_2_. For studies of C3‐mediated effects in cells, the pictures of the cells were taken after the indicated incubation periods with the toxins using an Axiovert 40CFl microscope from Zeiss (Oberkochen, Germany) connected to a ProgRes C10 CCD camera from Jenoptik (Jena, Germany). For internalization studies into cells, images were obtained using a LSM 710 laser scanning confocal microscope (LSCM) system (Zeiss, Germany) coupled to XL‐LSM 710 S incubator and equipped with a 63× oil immersion objective. The acquired images were processed with ImageJ software (NIH, Bethesda). For in vivo experiments, A549‐Red‐Fluc cells (Perkin Elmer, BW119266) were cultured in RPMI 1640 supplemented with 10% heat‐inactivated FCS and puromycin (2 µg mL^−1^).

*Cellular Uptake Studies of Transport Proteins, SST(N)‐Avi*: A549 cells were precultured in full growth medium and seeded at 6 500 cells per well in a white 96‐well (half‐area) plate. The cells were left to adhere overnight at 37 °C, 5% CO_2_. The media were removed and different concentrations of the transporters SST(N)‐Avi (50 × 10^−9^, 100 × 10^−9^, 200 × 10^−9^, and 500 × 10^−9^ m) in 50 µL DMEM were added into each well. Avi was added as a control. The treated cells were subsequently incubated separately for 4 h at 37 °C, 5% CO_2_. After incubation, the cells were washed (three times) with Dulbecco's phosphate‐buffered saline (PBS) buffer to remove nonspecific binding followed by incubating the cells for a further 24 h in 50 µL per well of cell lysis buffer. Emission measurements (λ_ex_ = 558 nm, λ_em_ = 585 nm) were recorded using a TECAN M1000 microplate reader to determine the uptake efficiencies. The values were given as mean ± standard deviation (SD) (*n* = 4), and data were analyzed by one‐way analysis of variance with Bonferroni correction for multigroup comparison at *p < 0.05, NS: not significant with Origin Pro 9.1.

*Expression and Purification of the Cys‐Mutant C3bot1‐A1C*: To insert a cysteine mutation, a mutant of *C. botulinum* C3bot1‐exoenzyme by site‐directed mutagenesis using appropriate PCR primers was constructed. The first amino acid Ala‐1 was replaced by cysteine. Afterward, the enzymatically active C3bot mutant C3bot1‐A1C (Cys‐C3) was overexpressed as glutathione S‐transferase (GST)‐tagged protein and purified by affinity chromatography. *Escherichia coli* BL21 transformed with pGEX2T‐Cys‐C3bot1 was grown in Luria‐Bertaini (LB) medium at 37 °C to an optical density of 0.6–0.8. The LB medium was added with 100 µg mL^−1^ ampicillin. After reaching the desired optical density, protein expression was induced by adding 200 × 10^−6^ m isopropyl‐β‐d‐thiogalactopyranoside and the cultures incubated overnight at 29 °C. The bacteria were harvested by centrifugation (5000 rpm, 10 min, 4 °C) and resuspended in lysis‐buffer containing 10 × 10^−3^ m NaCl, 20 × 10^−3^ m Tris‐HCl, 1% Triton X‐100, 1 × 10^−3^ m phenylmethylsulfonyl fluoride, pH 7.4. After harvesting, the bacteria were disrupted by sonification, cellular debris were centrifuged for 10 min at 12 000 rpm and 4 °C, and the clear supernatant was incubated overnight at 4 °C glutathione‐agarose beads (Macherey‐Nagel, Düren, Germany). After incubation, the toxin–bead mixture was centrifuged for 5 min at 2200 rpm and 4 °C, the beads were then washed two times with wash buffer containing 150 × 10^−3^ m NaCl, 20 × 10^−3^ m Tris, pH 7.4 and one time with PBS. The bound toxin was then incubated with thrombin (20 NIH‐units L^−1^ bacteria culture) for 1 h at room temperature (RT) to cleave the GST‐tag. The toxin‐containing supernatant was then obtained by centrifugation for 30 s at 4 °C and 10 000 rpm. Thrombin was removed by incubation of the supernatant with benzamidine beads (GE Healthcare, München, Germany) for 10 min at 21 °C. The purity of the isolated toxin was checked by SDS‐PAGE.

*Synthesis of Biotinylmaleimide with Hydrazone Linkage (3)*: Compound 1 (177 mg, 0.48 mmol, 3 eq.) and compound 2 (40 mg, 0.1506 mmol, 1 eq.) were dissolved in 3 mL of anhydrous MeOH under argon atmosphere. Thereafter, catalytic amount of trifluoroacetic acid (1 µL) was added and the resulting reaction mixture was stirred at RT for 24 h. The solvent was removed in vacuo and the residue was purified by column chromatography using 10% MeOH in CHCl_3_ to afford 69 mg with 88% yield. ^1^H‐NMR (500 MHz, CD_3_OD): δ = 1.48 (m, 2H), 1.57‐1.88 (m, 7H), 1.98 (br, 2H), 2.11‐2.44 (m, 6H), 2.71 (d, 1H, *J* = 12.7 Hz), 2.94 (d, 1H, *J* = 12.7 Hz), 3.22 (m, 1H), 3.36 (m, 2H), 3.63 (m, 2H), 4.33 (m, 1H), 4.51 (m, 1H), 6.83 (s, 2H) ppm. ^13^C‐NMR (125 MHz, CD_3_OD): δ = 16.54, 23.53, 26.62, 29.53, 29.80, 34.59, 35.68, 38.26, 38.41, 38.89, 41.03, 56.96, 61.63, 63.41, 127.34, 135.45, 162.15, 165.93, 172.56, 176.20 ppm. Liquid chromatography–mass spectrometry (electrospray ionization): *m*/*z* = 493 [M+H]^+^, 515 [M+Na]^+^ (calcd. mass: 492.22, formula: C_22_H_32_N_6_O_5_S).

*Biotinylation of C3 (B‐C3)*: Recombinant cysteine mutant of C3 was expressed and purified in *E. coli* BL21 as described in Expression and Purification of the Cys‐Mutant C3bot1A1C. HEPES (60 µL, 100 × 10^−3^ m, pH 7.4) of *N*‐(2‐hydroxyethyl)piperazine‐29‐(2‐ethane‐sulfonic acid) (HEPES) (100 × 10^−3^ m, pH 7.4) and 30 µL of the solution of compound **3** (5 mg mL^−1^ in DMF, 30 mol. eq.) were added to the Cys‐C3 solution (200 µg, 8 nmol, 1 mol. eq.) sequentially. The reaction mixture was shaken at RT for 3 h and purified by rigorous ultrafiltration with 3× buffer (molecular weight cut‐off (MWCO) = 10 kDa, buffer: 25 × 10^−3^ m HEPES, pH 7.4) to yield B‐C3 (208 µL, 0.7 mg mL^−1^, 73% yield). The concentration of B‐C3 was determined using a fluorescamine‐based fluorescence assay (λ_ex_ = 365 nm, λ_em_ = 480 nm) with bovine serum albumin as reference. The labeling of C3 was confirmed by the absence of free (unmodified) thiol groups in C3 using a 4,4′‐dithiodipyridine absorbance assay with cysteine as standard. Analysis was performed at λ_abs_ = 324 nm.

*Preparation of SST3‐Avi and SST‐Avi‐C3*: Avidin (5 mg, 1 mol. eq.) was dissolved in 5 mL of sodium bicarbonate buffer (20 × 10^−3^ m, pH 7.4) and fluorescein isothiocyanate (1 mg mL^−1^ in DMF, 90 µL, 3 mol. eq.) was added. The reaction mixture was stirred overnight and dried via lyophilization. The mixture was purified by size exclusion chromatography using Sepharose G‐25 matrix to afford 5 mg of fluorescein labeled avidin (FITC‐Avi) with quantitative yield. FITC‐Avi (1 mg, 1 mol. eq.) and 0.11 mg of B‐SST (3 mol. eq.) were dissolved in 1 mL of phosphate buffer (20 × 10^−3^ m, pH 7.4). The reaction mixture was shaken for 1 h and purified by size exclusion chromatography using Sephadex G‐25 matrix to afford SST3‐Avi quantitatively. 140 µL of B‐C3 (0.7 mg mL^−1^, 1 mol. eq.) and 29 µL of B‐SST (2 mg mL^−1^ in 25 × 10^−3^ m HEPES, pH 7.4, 3 mol. eq.) were added to 52 µL of FITC‐Avi (5 mg mL^−1^ in 25 × 10^−3^ m HEPES, pH 7.4, 1 mol. eq.) sequentially. The reaction mixture was shaken for 30 min and purified by rigorous ultrafiltration with 3× buffer (MWCO = 30 kDa, buffer: 25 × 10^−3^ m HEPES, pH 7.4) to yield SST‐Avi‐C3 quantitatively. The concentration of SST3‐Avi‐C3 for application was determined by linear calibration against FITC‐Avi at λ_abs_ = 495 nm. Successful bioconjugation was also verified by SDS‐PAGE analysis with and without heating (see the Supporting Information). The stability of the construct in different buffers and human serum was investigated (see the Supporting Information). Based on this experience, SST3‐Avi‐C3 is stable in storage at 4 °C in 25 × 10^−3^ m HEPES buffer (pH 7.4) for up to 1 year.

*Internalization of SST3‐Avi into Different Cancer Cell Types*: SSTR2‐positive A549 and LiSa‐2 cells as well as SSTR2‐negative SK‐UT1‐cells were seeded in 8‐well plates (ibidi GmbH, Munich, Germany) with a density of 30 000 cells per well in 300 µL. The cells were incubated at 37 °C and 5% CO_2_ for 24 h. SST3‐Avi (fluorescein‐labeled) with a final concentration of 400 × 10^−9^ m was added to a total volume of 300 µL. As control, cells were treated only with DMEM. After 24 h, the cells were washed with PBS and reconstituted with complete DMEM and imaging was performed using LSCM.

*Analysis of the Effects Mediated by C3 on A549 Cells*: A549 cells were seeded in 96‐well plastic dishes and were incubated with increasing amounts of C3 (200 × 10^−9^, 400 × 10^−9^, 600 × 10^−9^, 1000 × 10^−9^, and 1500 × 10^−9^ m) in DMEM medium at 37 °C and 5% CO_2_. For control, cells were left untreated. After 24 h incubation, phase contrast pictures were taken with a Zeiss Axiovert microscope (200× magnification); representative pictures were shown. The effects on the cells were determined via quantification of degree of change in cells morphology. For quantitative analysis, the percentage of A549 cells showing C3‐morphology was determined from three independent samples treated in the same way (*n* = 3, values were given as mean ± SD, average number of cells per well: 130, blinded evaluation of cell morphology).

*Detection of Cell‐Associated C3*: A549 cells were seeded overnight in a 24‐well plate and then incubated with 350 × 10^−9^ m SST3‐Avi‐C3, 350 × 10^−9^ m C3, 350 × 10^−9^ m B‐C3, or were left untreated. After 24 h, cells were washed several times, lysed in hot 2.5 × Lämmli‐buffer + dithiothreitol (DTT) and heated at 95 °C for 10 min. Next, lysates were separated by SDS‐PAGE, transferred onto a nitrocellulose membrane via Western blot, and membrane was blocked with 5% dry milk in PBS‐T for 30 min. Cell‐associated C3 was stained with a rabbit‐anti‐C3‐antibody (1:10 000 in PBS‐T for 30 min) in combination with a goat antirabbit‐antibody (1:2500 in PBS‐T for 30 min), and subsequently visualized by chemiluminescence reaction using Immobilon Western Chemiluminescent HRP Substrate from Millipore (Schwalbach, Germany) according to the manufacturer's instruction. Equal protein loading was confirmed by actin staining with a mouse–antiactin‐antibody (1:10 000 in PBS‐T for 30 min) in combination with a chicken–antimouse‐HRP‐antibody (1:2500 in PBS‐T for 30 min) and subsequent chemiluminescence reaction.

*Analysis of the Effects Mediated by SST3‐Avi‐C3 on A549 Cells*: For the cytotoxicity experiments, cells were seeded in 96‐well plastic dishes and were incubated with SST3‐Avi‐C3 in DMEM medium at 37 °C and 5% CO_2_. As control, cells were incubated with SST3‐Avi‐C3, SST3‐Avi, or C2IN‐C3/C2IIa, or were left untreated. After the indicated incubation times, pictures of the cells were acquired. The effects on cell morphology were determined by counting the cells showing the characteristic C3‐morphology in a blinded manner by two independent investigators. The values were given as mean ± SD (*n* = 3).

*In Vitro ADP‐Ribosylation of Rho in A549 Cells*: SSTR2‐positive A549 cells were incubated together with either SST3‐Avi‐C3, SST3‐Avi, C2IN‐C3/C2IIa, or were left untreated for 24 h, harvested in ADP‐ribosylation buffer containing 1 × 10^−3^ m DTT, 5 × 10^−3^ m MgCl_2_, 1 × 10^−3^ m ethylenediaminetetraacetic acid, 20 × 10^−3^ m Tris‐HCl, pH 7.5, and lysed using a 25 gauge needle. Equal amounts of protein were incubated with 10 × 10^−3^ m biotin‐labeled NAD^+^ and 300 ng C3 for 30 min at 37 °C. The enzyme reaction was stopped by the addition of SDS sample buffer and subsequent heating at 95 °C for 10 min. Followed by SDS‐PAGE and Western blot transfer onto a nitrocellulose membrane, the biotin‐labeled ADP‐ribosylated Rho proteins were detected with peroxidase‐coupled streptavidin (1:2500) and subsequent chemiluminescence reaction according to the manufacturer's instruction. Comparable protein loading was confirmed by actin staining as described in Detection of Cell‐Associated C3.

*Internalization of SST3‐Avi‐C3 into Mammalian Cells*: For the immunocytochemical analysis A549 cells were seeded in 8‐well plates (ibidi GmbH, Munich, Germany) with a density of 30 000 cells per well in 300 µL. The cells were incubated at 37 °C and 5% CO_2_ overnight in DMEM medium. Then cells were incubated for 24 h with SST3‐Avi‐C3, SST3‐Avi, or left untreated as control. Next, cells were washed, fixed with 4% paraformaldehyde, permeabilized using 0.4% Triton X‐100, and blocked with 5% dry milk powder in PBS‐T. To visualize C3, the toxin was stained with a rabbit‐anti‐C3‐antibody (1:2000 in 5% dry milk in PBS‐T for 30 min) in combination with an Alexa Fluor 647‐coupled goat–antirabbit secondary antibody (1:2000 in 5% dry milk in PBS‐T). The SST3‐Avi transporter was FITC labeled. Imaging was then performed using an LSCM.

*CAM In Vivo Xenografts*: 1 × 10^6^ A549 cells were grafted in medium/Matrigel (1:1, v/v) onto the CAM of chick eggs 8 d after fertilization. One day later, SST3‐Avi‐C3 (350 × 10^−9^ m) or controls/chemotherapeutics (NaCl 0.9%, SST3‐Avi (400 × 10^−9^ m), B‐C3 (350 × 10^−9^ m; Figure S12, Supporting Information), bevacizumab (34 × 10^−6^ m), and doxorubicin (100 × 10^−6^ m) were topically applied in 20 µL. After 24 h, tumor xenografts were collected and embedded in paraffin for immunohistological staining (i.e., hematoxylin and eosin (HE)) to reveal morphology; Ki‐67 proliferation marker to identify the growth fraction within the tumor xenografts; TUNEL staining, terminal deoxynucleotidyl transferase (Tdt)‐mediated deoxyuridine triphosphate (dUDP)‐biotin nick end labeling, to tag nuclei of apoptotic cells; desmin staining to identify new blood vessel formation. To calculate the proportion of marker positive and negative cells, 200–790 cells per tumor were evaluated.

## Conflict of Interest

The authors declare no conflict of interest.

## Supporting information

Supplementary
