# Supplementary material for: Boosting Antitumor Drug Efficacy with Chemically Engineered Multidomain Proteins
Source: Adv Sci (Weinh). 2018 Jun 14;5(8):1701036. doi: 10.1002/advs.201701036 (PMC6097141; doi:10.1002/advs.201701036)
Supplement: Supplementary file 1 — Supplementary [file ADVS-5-1701036-s001.pdf]

Copyright WILEY-VCH Verlag GmbH & Co. KGaA, 69469 Weinheim, Germany, 2018.

ADVANCED SCIENCE
Open Access

Supporting Information

for *Adv. Sci.,* DOI: 10.1002/advs.201701036

Boosting Antitumor Drug Efficacy with Chemically Engineered Multidomain Proteins

Seah Ling Kuan, Stephan Fischer, Susanne Hafner, Tao Wang, Tatiana Syrovets, Weina Liu, Yu Tokura, David Yuen Wah Ng, Andreas Riegger, Christina Förtsch, Daniela Jäger, Thomas F. E. Barth, Thomas Simmet,* Holger Barth,* and Tanja Weil**

Copyright WILEY-VCH Verlag GmbH & Co. KGaA, 69469 Weinheim, Germany, 2016.

Supporting Information

Boosting Anti-Tumor Drug Efficacy with Chemically Engineered Multidomain Proteins

Seah Ling Kuan,[†,1,2] Stephan Fischer,[†,2,3] Susanne Hafner,[†,4] Tao Wang,[2,5] Tatiana Syrovets,[4] Weina Liu,[1,2] Yu Tokura,[1,2] David Yuen Wah Ng,[1,2] Andreas Riegger,[2] Christina Förtsch,[3] Daniela Jäger,[6] Thomas F. E. Barth,[6] Thomas Simmet*,[4] Holger Barth*,[3] Tanja Weil*.[1,2]*

[†]contributed equally

[1] Max-Planck Institute for Polymer Research, Ackermannweg 10, 55128 Mainz, Germany

[2] Institute of Inorganic Chemistry I – Ulm University, Albert-Einstein-Allee 11, 89081 Ulm, Germany

[3]Institute of Pharmacology and Toxicology, University of Ulm Medical Center, Albert-Einstein-Allee 11, 89081 Ulm, Germany

[4]Institute of Pharmacology of Natural Products and Clinical Pharmacology, Ulm University, Helmholtzstraße 20, 89081 Ulm, Germany

[5]School of Materials Science and Engineering, Southwest Jiaotong University, 610031, Chengdu, P.R. China

[6]Institute of Pathology, Ulm University, Albert-Einstein-Allee 23, 89070 Ulm, Germany

E-mail: kuan@mpip-mainz.mpg.de, weil@mpip-mainz.mpg.de, holger.barth@uni-ulm.de, thomas.simmet@uni-ulm.de

General information and methods. All solvents and reagents were bought from commercial sources (Merck, Sigma Aldrich, Fluka and Thermo Scientific) and were used directly without further purification. Somatostatin acetate was purchased from GLBiochem. The extent of reaction was monitored by thin layer chromatography using Merck 60 F254 pre-coated silica on aluminum using appropriate stains ($KMnO_4$, ninhydrin, iodine). Flash column chromatography was carried out on Acros Organics silica gel 0.035 mm - 0.070 mm, 60 Å. ^{1}H-NMR and ^{13}C-NMR were recorded using a Bruker DRX 400 MHz Fourier transform spectrometer using $CDCl_3$. Chemical shifts are reported as parts per million referenced with respect to the residual solvent peak. ESI-MS was recorded using Shimadzu LC-MS 2020. N-(2-aminoethyl)maleimide trifluoroacetate salt[1] and biotinylated-somatostatin (B-SST)[2] were synthesized as previously reported. Amicon Ultra (MWCO 10 and 30 kDa) from Millipore Corporation with an Eppendorf centrifuge 5810R were used for ultrafiltration. Mass spectra were acquired on a Bruker Daltonics Reflex III MALDI TOF; the absorbance and emission were measured on microplate readers (Tecan Infinite M1000 PRO or Tecan Infinite M200) and UV/VIS measurements were performed using a Nanodrop 2000 spectrophotometer. AFM measurements were performed using a Bruker Dimension FastScan Bio.

Optimization of biotinylated somatostatin (B-SST)-avidin binding. Native avidin (Novabiochem) and B-SST(43) (0-6 eq.) were dissolved in phosphate buffer (50 mM, pH 7.4) to afford 1 mg ml^{-1} avidin solutions with different equivalents of B-SST. The respective solutions (100 µl) and HABA solution (1 mg ml^{-1} in DMSO, 2 µl) were introduced into a UV-star® flat bottom 384-well plate (Greiner bio-one, Frickenhausen, Germany). The mixture was mixed and the absorbance spectrum (250 nm–650 nm) was measured.

Preparation of transport proteins SST1-Avi, SST2-Avi, SST3-Avi, SST4-Avi. 10 mg of avidin were dissolved in 10 ml of phosphate buffer (50 mM, pH 7.4) and rhodamine B isothiocyanate (1 mg ml^{-1} in DMF, 406 µl) was added. The reaction mixture was stirred overnight and dried *via* lyophilization. The mixture was purified by size exclusion chromatography using Sephadex G-25 matrix to afford 10 mg of rhodamine-labeled avidin (Rh-Avi) with quantitative yield. Rh-Avi (2 mg) and different equivalents of B-SST (1-3 eq. and 6 eq.) were dissolved in phosphate buffer (50 mM, pH 7.4) separately and the reaction mixtures were incubated for 1 h. The mixtures were purified by size exclusion chromatography using Sephadex G-25 matrix to afford SST1-Avi, SST2-Avi, SST3-Avi, SST4-Avi with quantitative yields.

Cell culture. A549, SK-UT-1 (DSMZ, German Collection of Microorganisms and Cell Cultures) and LiSa-2(50) cells were cultivated at 37°C and 5% CO_2 in Dulbecco's Modified

Eagle Medium (DMEM, high glucose). Media contained 10% heat inactivated FCS, L-glutamate (2 mM) and 100 U ml^{-1} penicillin and 100 µg ml^{-1} streptomycin. Cells were routinely trypsinized and reseeded twice per week. For the experiments, cells were seeded in plastic dishes and incubated with the respective compounds in the medium at 37°C and 5% CO_2. For studies of C3-mediated effects in cells, the pictures of the cells were taken after the indicated incubation periods with the toxins using an Axiovert 40CFl microscope from Zeiss (Oberkochen, Germany) connected to a progress C10 CCD camera from Jenoptik (Jena, Germany). For internalization studies into cells, images were obtained using a LSM 710 laser scanning confocal microscope (LCSM) system (Zeiss, Germany) coupled to XL-LSM 710 S incubator and equipped with a 63x oil immersion objective. The acquired images were processed with ImageJ software (NIH, Bethesda). For *in vivo* experiments, A549-Red-Fluc cells (Perkin Elmer, BW119266) were cultured in RPMI 1640 supplemented with 10% heat-inactivated FCS and puromycin (2 µg ml^{-1}).

Cellular uptake studies of transport proteins, SST(N)-Avi. A549 cells were pre-cultured in high glucose DMEM medium fortified with 10% fetal bovine serum, 1% penicillin/streptomycin and seeded at 6,500 cells/well in a white 96-well (half-area) plate. The cells were left to adhere overnight at 37°C, 5% CO_2. The media were removed and different concentrations of the transporters SST(N)-Avi (50 nM, 100 nM, 200 nM, 500 nM) in 50 µl DMEM were added into each well. Avi was added as a control. The treated cells were subsequently incubated separately for 4 h at 37°C, 5% CO_2. After incubation, the cells were washed (3 times) with Dulbecco's PBS buffer to remove non-specific binding followed by incubating the cells for a further 24 h in 50 µl per well of cell lysis buffer. Emission measurements (λ_{ex} = 558 nm, λ_{em} = 585 nm) were recorded using TECAN M1000 microplate reader to determine the uptake efficiencies. The values were given as mean ± SD (n=4) and data were analyzed by one-way analysis of variance (ANOVA) with Bonferroni correction for multi-group comparison at *$p<0.05$, NS = not significant. with Origin Pro 9.1.

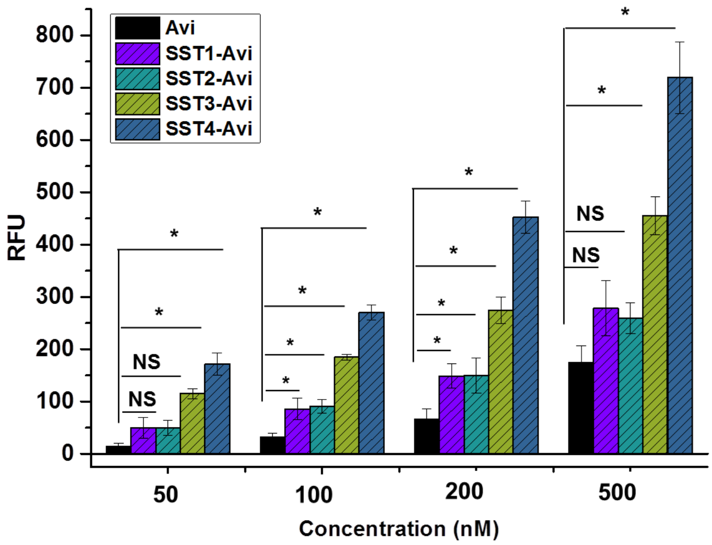

Figure S1. Full statistical analysis of the cellular uptake of SST(N)-Avi transporters into A549 cells and concentration dependency shown in Fig. 2b in main text. The values are given as mean ± SD. Data were analyzed by one-way ANOVA with Bonferroni correction for multi-group comparison at *$p<0.05$, NS = not significant.

Internalization of the transporter SST4-Avi into mammalian cells. A549 cells were seeded at a density 30,000 cells/well in a µ-Slide 8-well chambered coverslip (ibidi, Munich, Germany) in 300 µl DMEM medium with 10% FCS. The cells were cultured overnight to allow adhesion at 37°C, 5% CO_2. Subsequently, the medium was removed and 2 µM of Avi and SST4-Avi were added, respectively. The cells were then further incubated for 4 h at 37°C, 5% CO_2. Before imaging, cells were washed with DMEM medium for 3 times. Cell membrane was stained with 0.5 µl of CellMask Deep Red (0.5 mg ml^{-1}, $\lambda_{ex/em}$: 649/666) for 5 min. The live cell imaging was performed using a LSM 710 laser scanning confocal microscope system (Zeiss, Germany) coupled to an XL-LSM 710 S incubator and equipped with a 63x oil immersion objective. The emission of SST4-Avi was recorded using a 525-759 nm filter and a 514 nm argon laser for excitation. The acquired images were processed with ZEN 2011 software (Figure S2).

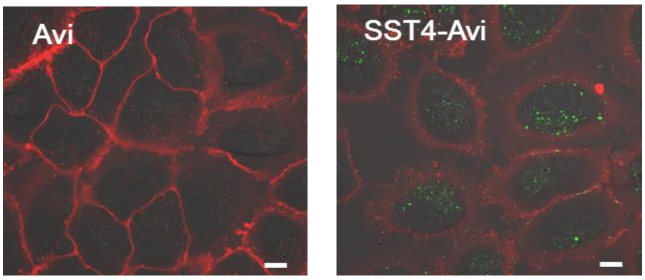

Figure S2. Confocal images of A549 cells incubated with 2 µM Avi or SST4-Avi (green) for 4 h. Cell membranes were stained (red) to confirm internalization. Scale bar: 10 µm.

Functional assay for SST agonist activity against SSTR2. Cell-type selectivity was demonstrated by applying SST4-Avi to wild type CHO-K1/Gα15 40 cells and CHO-K1/Gα15/SSTR2 cells overexpressing SSTR2 for a functional calcium flux assay conducted by GenScript. The assay was performed in the agonist mode in duplicate.

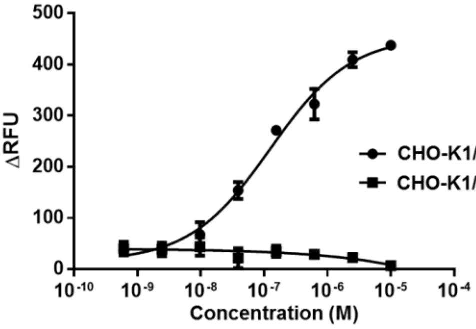

Figure S3. Calcium release induced by incubation of SST4-Avi (EC_{50} = 125 nM) with CHO-K1 cells overexpressing SSTR2 (circles) and wild type CHO-K1 cells (squares) was measured to confirm SSTR2-mediated internalization. RFU = relative fluorescence units.

Calcium flux assay (Genscript) to measure calcium release induced by the agonistic interactions between SSTR2 and its ligand showed that SST4-Avi induced significant receptor activation in CHO-K1/Gα15 cells overexpressing SSTR2 already at low concentrations (EC_{50} of 125 nM), whereas only a basal signal was observed for all concentrations of SST4-Avi in the case of the SST receptor-deficient, wild type CHO-K1/Gα15 cells. The result suggested that the interaction of the SST-Avi conjugates with the cells was specifically mediated by the SST-2 ligand (Fig. S3).

Internalization of SST3-Avi into different cancer cell types. SSTR2-positive A549 and LiSa-2 cells as well as SSTR2-negative SK-UT1-cells were seeded in 8-well plates (ibidi GmbH, Munich, Germany) with a density of 30,000 cells per well in 300 µl. The cells were incubated at 37°C and 5% CO_2 for 24 h. SST3-Avi (fluorescein-labeled) with a final concentration of

400 nM was added to a total volume of 300 µl. As control, cells were treated only with DMEM. After 24 h, the cells were washed with PBS and reconstituted with complete DMEM and imaging was performed using LSCM.

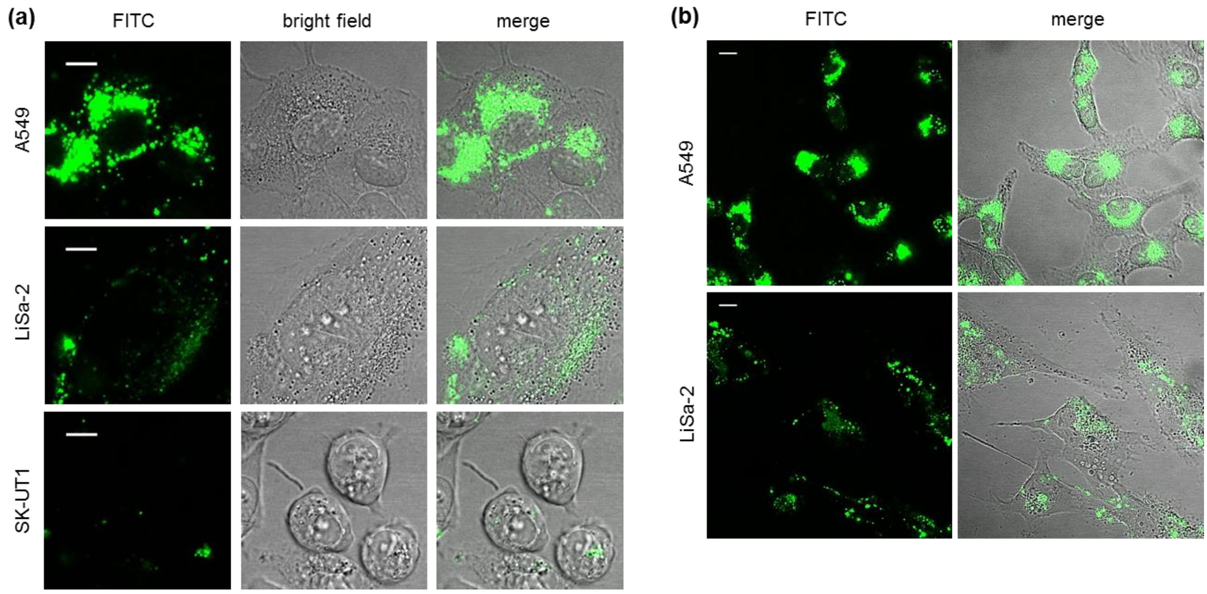

Fig S4. LSM analysis demonstrates the selective uptake of SST3-Avi-transporter into SSTR2-positive human tumor cell lines. (**a**) A549, LiSa-2 (both SSTR2-positive) and SK-UT1 (SSTR2-negative) cells grown in eight well µ-Slide-plates (ibiTreat) were incubated at 37 °C with SST3-Avi (400 nM). After 24 h, cells were washed and the uptake of FITC-labelled SST3-Avi was analyzed by immunofluorescence microscopy. (**b**) Overview pictures from A549 and LiSa-2 cells showing internalized SST3-Avi in each cell of both SSTR2-positive cell lines. Scale bars correspond to 10 µm.

Expression and purification of the Cys-mutant C3bot1-A1C. To insert a cysteine-mutation, we constructed a mutant of *C. botulinum* C3bot1-exoenzyme by site-directed mutagenesis using appropriate PCR primers. By doing so, we replaced Ala-1 by cysteine. Afterwards, the enzymatically active C3bot mutant C3bot1-A1C (Cys-C3) was overexpressed as GST-tagged protein and purified by affinity chromatography. *E. coli* BL21 transformed with pGEX2T-Cys-C3bot1 were grown in Luria-Bertani medium at 37°C to an optical density of 0.6 – 0.8. The LB-medium was added with 100 µg ml^{-1} ampicillin. After reaching the desired optical density, protein expression was induced by adding 200 µM isopropyl-β-D-thiogalactopyranoside (IPTG) and the cultures incubated overnight at 29°C. The bacteria were harvested by centrifugation (5000 rpm, 10 min, 4°C) and resuspended in lysis-buffer containing 10 mM NaCl, 20 mM Tris-HCl, 1% Triton X-100, 1 mM PMSF, pH 7.4. After harvesting, the bacteria

were disrupted by sonification, cellular debris were centrifuged for 10 min at 12000 rpm and 4°C and the clear supernatant was incubated overnight at 4°C glutathione-agarose beads (Macherey-Nagel, Düren, Germany). After incubation, the toxin-bead-mixture was centrifuged for 5 min at 2200 rpm and 4°C, the beads were then washed two times with wash buffer containing 150 mM NaCl, 20 mM Tris, pH 7.4 and one time with PBS. The bound toxin was then incubated with thrombin (20 NIH-units l^{-1} bacteria culture) for 1 h at room temperature to cleave the GST-tag. The toxin-containing supernatant was then obtained by centrifugation for 30 s at 4°C and 10000 rpm. Thrombin was removed by incubation of the supernatant with benzamidine beads (GE Healthcare, München, Germany) for 10 min at 21°C. The purity of the isolated toxin was checked by SDS-PAGE.

Expression and characterization of C3bot1-A1C. The cysteine-C3 mutant was generated by site-directed mutagenesis with the pGEX2T-C3bot1 (genes kindly provided by Dr. Klaus Aktories, Freiburg, Germany) as a template and the respective oligonucleotides using the Quick Change II XL site-directed mutagenesis kit (Agilent Technologies) according to manufacturer's instructions. Two complementary synthetic oligonucleotides were needed (only one of the two is indicated). C3bot1-A1C:

5′-TCCGGTGGTGGTGGTGGATCCTGTTATTCAAATACTTACCAGGAG-3′.

The mutated plasmid was transformed into *E. coli* BL21 competent cells and the presence of the mutation was confirmed by DNA sequencing. C3bot1-A1C was expressed as recombinant glutathione S-transferase-fusion protein in *E.coli* containing the respective DNA plasmid (C3bot1-A1C gene). Protein expression and the enzymatic activity of the fusion proteins were confirmed via SDS-PAGE and Western blotting (Fig. S5a, S5b).

J774A.1 macrophage-like cells (obtained from Dr. Singh Chhatwal, Braunschweig, Germany) were cultivated at 37°C and 5% CO_2 in Dulbecco's modified Eagle medium (DMEM), containing 10% heat-inactivated (30 min at 56°C) FCS. The medium contained L-glutamate (4 mM), penicillin (100 U ml^{-1}) and streptomycin (100 µg ml^{-1}). Cells were split at least twice per week. Cells were incubated with C3bot1-A1C, C3bot1 or medium only at a final concentration of 3 µg ml^{-1} for 6 h and C3-induced morphological changes were recorded (Fig. S5c).

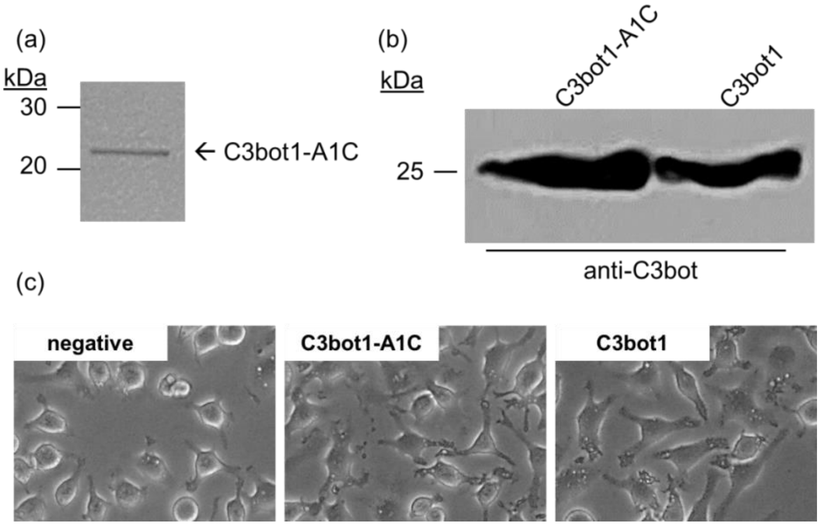

Figure S5. Characterization of C3bot1-A1C. (**a**) Coomassie blue staining of the expressed and purified mutant C3bot1-A1C after SDS-PAGE. (**b**) 300 ng of C3bot1-A1C as well as 300 ng of C3bot1 for control were subjected to SDS-PAGE followed by Western blotting. Expression of the protein was confirmed by detecting with a specific antibody against C3bot1. (**c**) J774A.1 macrophage-like cells were treated for 6 h with either C3bot1-A1C (3 μg ml^{-1}), C3bot1 (3 μg ml^{-1}) or were left untreated (control) and C3-induced morphological changes were recorded to demonstrate biological activity of the proteins.

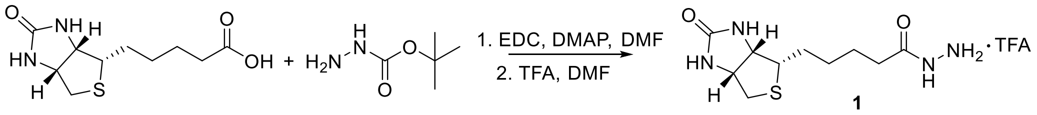

Scheme S1. Synthesis of compound **1**.

Synthesis of biotinylhydrazine linker (1). Biotin (200 mg, 0.82 mmol, 1 eq.), EDC (188 mg, 0.98 mmol, 1.2 eq.) and DMAP (12 mg, 98 μmol, 0.12 eq.) were dissolved in 5 ml of anhydrous DMF at 0°C under argon atmosphere and thereafter *tert*-butyl carbazate (130 mg, 0.98 mmol, 1.2 eq.) was added. The reaction mixture was stirred at RT overnight and the solvent was removed by high vacuum. The residue was purified by column chromatography by using eluting solvents 10% MeOH in $CHCl_3$ to afford Boc protected biotinylhydrazine. This compound was further dissolved in 4 ml of DCM and 1 ml of TFA was added. The resulting mixture was stirred overnight and the solvents were removed by high vacuum to afford 274 mg of biotinylhydrazine 1 (0.74 mmol, 90% yield). ^{1}H NMR (400 MHz, D_2O) δ 1.24-1.43 (m, 2H), 1.52-1.71 (m, 4H), 2.28 (t, J = 7.2 Hz, 2H), 2.76 (d, J =13.2 Hz, 1H), 2.98 (dd, J = 13.2, 4.9 Hz, 1H), 3.37 (m, 1H), 4.41 (m, 1H), 4.59 (m, 1H); ^{13}C NMR (100 MHz, D_2O) δ 25.4, 28.5, 28.7, 33.7, 40.3, 56.0, 60.9, 62.8, 166.0, 176.4.

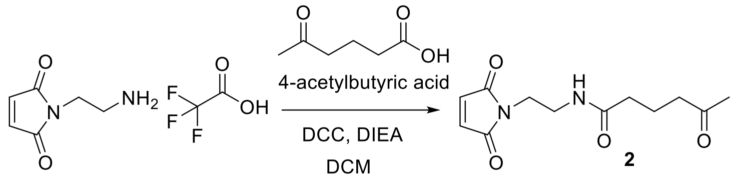

Scheme S2. Synthesis of compound **2**.

Synthesis of N-(2-(2,5-dioxo-2,5-dihydro-1H-pyrrol-1-yl)ethyl)-5-oxohexanamide linker (2). 4-acetylbutyric acid (155 mg, 1.18 mmol, 1.2 eq.) and *N*-(2-aminoethyl)maleimide trifluoroacetate salt (250 mg, 0.98 mmol, 1 eq.) were dissolved in 5 ml of anhydrous DCM at 0°C under argon atmosphere. DIEA (300 µl, 1.77 mmol, 1.8 eq.) and DCC (405 mg, 1.97 mmol, 2 eq.) were added sequentially, and the resulting reaction mixture was stirred overnight at RT. The precipitate was filtered and the solvent was removed by high vacuum. The residue was purified by column chromatography by using eluting solvents 3% MeOH in $CHCl_3$ to afford 240 mg of the product 2 (0.95 mmol, 97% yield). ^{1}H NMR (400 MHz, $CDCl_3$) δ 1.80 (m, 2H), 2.11 (m, 5H), 2.46 (t, *J* = 7.0 Hz, 2H), 3.41 (q, *J* = 5.5 Hz, 2H), 3.65 (t, *J* = 5.4 Hz, 2H), 6.70 (s, 2H).

Synthesis of biotinylmaleimide with hydrazone linkage (3). Compound 1 (177 mg, 0.48 mmol, 3 eq.) and compound 2 (40 mg, 0.1506 mmol, 1 eq) were dissolved in 3 ml of anhydrous MeOH under argon atmosphere. Thereafter, catalytic amount of TFA (1 µl) was added and the resulting reaction mixture was stirred at RT for 24 h. The solvent was removed *in vacuo* and the residue was purified by column chromatography using 10% MeOH in $CHCl_3$ to afford 69 mg with 88% yield. ^{1}H-NMR (500 MHz, CD_3OD): δ = 1.48 (m, 2H), 1.57-1.88 (m, 7H), 1.98 (br, 2H), 2.11-2.44 (m, 6H), 2.71 (d, 1H, *J* = 12.7 Hz), 2.94 (d, 1H, *J* = 12.7 Hz), 3.22 (m, 1H), 3.36 (m, 2H), 3.63 (m, 2H), 4.33 (m, 1H), 4.51 (m, 1H), 6.83 (s, 2H) ppm. ^{13}C-NMR (125 MHz, CD_3OD): δ = 16.54, 23.53, 26.62, 29.53, 29.80, 34.59, 35.68, 38.26, 38.41, 38.89, 41.03, 56.96, 61.63, 63.41, 127.34, 135.45, 162.15, 165.93, 172.56, 176.20 ppm. LC-MS (ESI): m/z = 493 $[M+H]^+$, 515 $[M+Na]^+$ (calcd. mass: 492.22, formula: $C_{22}H_{32}N_6O_5S$).

Biotinylation of C3 (B-C3). Recombinant cysteine mutant of C3 was expressed and purified in *E. coli* BL21 as described above. 60 µl of HEPES (100 mM, pH 7.4) and 30 µl of the solution of compound **3** (5 mg ml^{-1} in DMF, 30 mol. eq.) were added to the Cys-C3 solution (200 µg, 8 nmol, 1 mol. eq.) sequentially. The reaction mixture was shaken at RT for 3 h and purified by rigorous ultrafiltration with 3 x buffer (MWCO = 10 kDa, buffer: 25 mM HEPES, pH 7.4) to yield B-C3 (208 µl, 0.7 mg ml^{-1}, 73% yield). The concentration of B-C3 was determined using a fluorescamine-based fluorescence assay (λ_{ex} = 365 nm, λ_{em} = 480 nm) with BSA as reference. The labeling of C3 was confirmed by the absence of free (unmodified) thiol groups in C3 using a 4,4'-dithiodipyridine (4-DPS) absorbance assay with cysteine as standard. Analysis was

performed at λ_{abs} = 324 nm.

Preparation of SST3-Avi and SST-Avi-C3. 5 mg of avidin (1 mol. eq.) was dissolved in 5 ml of sodium bicarbonate buffer (20 mM, pH 7.4) and fluorescein isothiocyanate (1 mg ml^{-1} in DMF, 90 µl, 3 mol. eq.) was added. The reaction mixture was stirred overnight and dried *via* lyophilization. The mixture was purified by size exclusion chromatography using Sepharose G-25 matrix to afford 5 mg of fluorescein labeled avidin (FITC-Avi) with quantitative yield. 1 mg of FITC-Avi (1 mol. eq.) and 0.11 mg of B-SST (3 mol. eq.) were dissolved in 1 ml of phosphate buffer (20 mM, pH 7.4). The reaction mixture was shaken for 1 h and purified by size exclusion chromatography using Sephadex G-25 matrix to afford SST3-Avi quantitatively. 140 µl of B-C3 (0.7 mg ml^{-1}, 1 mol. eq.) and 29 µl of B-SST (2 mg ml^{-1} in 25 mM HEPES, pH 7.4, 3 mol. eq.) were added to 52 µl of FITC-Avi (5 mg ml^{-1} in 25 mM HEPES, pH 7.4, 1 mol. eq.) sequentially. The reaction mixture was shaken for 30 min and purified by rigorous ultrafiltration with 3 x buffer (MWCO = 30 kDa, buffer: 25 mM HEPES, pH 7.4) to yield SST-Avi-C3 quantitatively. The concentration of SST3-Avi-C3 for application was determined by linear calibration against FITC-Avi at λ_{abs} = 495 nm. Successful bioconjugation was also verified by SDS-PAGE analysis with and without heating (SI). The stability of the construct in different buffers and human serum was investigated (SI). Based on our experience, SST3-Avi-C3 is stable in storage at 4 °C in 25 mM HEPES buffer (pH 7.4) for up to one year.

Gel electrophoresis of SST3-Avi-C3. SDS-PAGE analysis was carried out using 8-16% Criterion™ TGX Stain-Free™ Protein Gel (Biorad). For routine purposes (determined empirically), samples (2 µM of SST3-Avi, SST3-Avi-C3 and B-C3) were treated with sample buffer at RT and at 95 °C respectively, prior to electrophoresis. Samples were analyzed without heating at RT to minimize avidin-biotin cleavage.

(1) With heating. 3 µl of loading dye (Invitrogen) and 1 µl of 1M DTT were added to 8 µl of protein solution. The mixture was heated for 5 min at 95 °C and cooled to RT for 5 min. 10 µl of the solution was loaded to the SDS-PAGE gel.
(2) Without heating. 4 µl of 50% glycerol solution was added to 8 µl of protein solution. 10 µl of the solution was loaded to the SDS-PAGE gel.

The gel was run using 1 l of 1X Tris-Glycine-SDS buffer with a constant 150 V for 50 min using AppliChem Protein Marker VI® protein ladders as a reference. The proteins were stained and visualized using Imperial™ Protein Stain (Thermo Scientific).

Atomic Force Microscopy of SST3-Avi-C3. 70 µl of a 10 µg ml^{-1} solution of protein substrate in MilliQ water was dropped onto freshly cleaved mica and absorbed for 5 min at RT. The sample was imaged using the liquid tapping mode on a Bruker Dimension FastScan Bio at the

Institute of Biophysics, Ulm University. The sample was scanned in ScanAsyst fluid mode with scan rates between 1 and 3 Hz. The sharpened pyramidal tips (ScanAsyst Fluid+, Bruker) were used for scanning and images were analyzed by using Gwyddion 2.38 software.

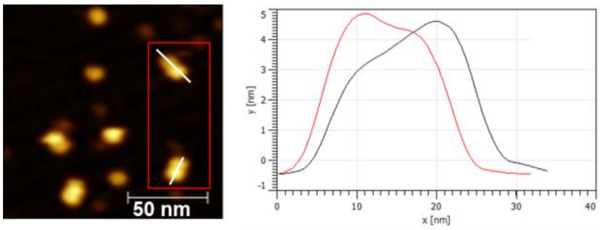

Figure S6. AFM image showing dimeric structures. A height profile analysis of representative dimers confirmed the formation of the heterodimeric conjugate, SST3-Avi-C3 (see inset).

Zetapotential measurements. A solution of 1 mg/mL avidin or 0.5 mg/mL unmodified C3 was prepared in 25 mM HEPES buffer and diluted 1:1 with 1 mM KCl. The zetapotential values were acquired using a Malvern Nano Zetasizer over min. 10 runs. As a control, the zetapotential of a solution with final concentration of 0.5 mg/mL human serum albumin (pI < 5) was determined.

Table S1. Zeta values of Avi, C3 and SST3-Avi-C3 using Malvern Zetasizer.

	Avi	C3	HSA
Zeta value (mV)	3.6	0.8	-12.4
Zeta deviation (mV)	5.2	4.0	5.4

Western blot analysis. The formation of SST3-Avi-C3 was confirmed using Western blot analysis and immunoblot detection with a streptavidin/peroxidase conjugate to detect the presence of B-C3.

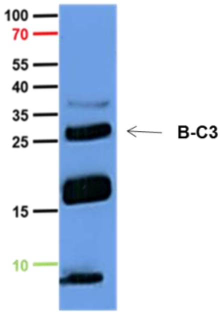

Figure S7. Immunoblot detection with a streptavidin/peroxidase conjugate by applying 350 nM of SST3-Avi-C3 with heating condition (see above protocol for gel electrophoresis) confirms the presence of B-C3 and formation of the conjugate.

Stability of SST3-Avi-C3 in buffer and cleavage at acidic pH. B-C3 was fluorescently labeled using BODIPY FL® NHS ester (Lumiprobe) and purified using Zeba spin desalting columns with MWCO 3 kDa (Thermo Scientific). BODIPY FL® was selected due to its stability in both neutral and acidic media. The fluorescently labeled B-C3 was preincubated as a solution in either 50 mM pH 4 acetate or 50 mM pH 7 HEPES buffer overnight at a concentration of 460 nM for the microscale thermophoresis (MST) measurements. SST3-Avi was prepared as described in the main text and a series of 1:1 dilutions (12 solutions) were prepared using MST buffer (50 mM TrisHCl pH 7.4, 150 mM NaCl, 10 mM $MgCl_2$, 0.05% Tween-20) with the highest concentration at 254 μM. 10 μl of B-C3 in pH 7 buffer was titrated against 10 μl of SST3-Avi (unlabeled) solutions and mixed homogenously. The solutions were then filled into Monolith NT premium coated capillaries (NanoTemper Technlogies GmbH) and the thermophoresis was measured using a Monolith NT.115 instrument (NanoTemper Technologies GmbH) at RT with 5s/30s/5s laser off/on/off, 30% LED power and 20% MST power. The average from two readings was obtained for the plots. The change in F_{norm} signal with increasing concentration of SST3-Avi is a clear indication of binding. Additional measurement was acquired after incubation up to 16 h to determine the stability of the SST3-Avi-C3 complex. The measured thermophoresis/T-jump signals were extracted and the curves were analyzed and fitted with the MST K_d function provided by NanoTemper Technologies GmbH (Fig. S8).

After 18 h, the solutions prepared above were aliquoted (5 μl) and 0.2 μl of 1 M HCl was added to each solution for acidification. MST measurements were obtained after 4 h and there was no binding observed (Fig. S8, blue), which indicated the acid-induced C3 release.

A similar procedure was repeated for B-C3 in pH 4 buffer as a negative control and no binding was observed upon incubation with SST3-Avi (Fig. S8, magenta).

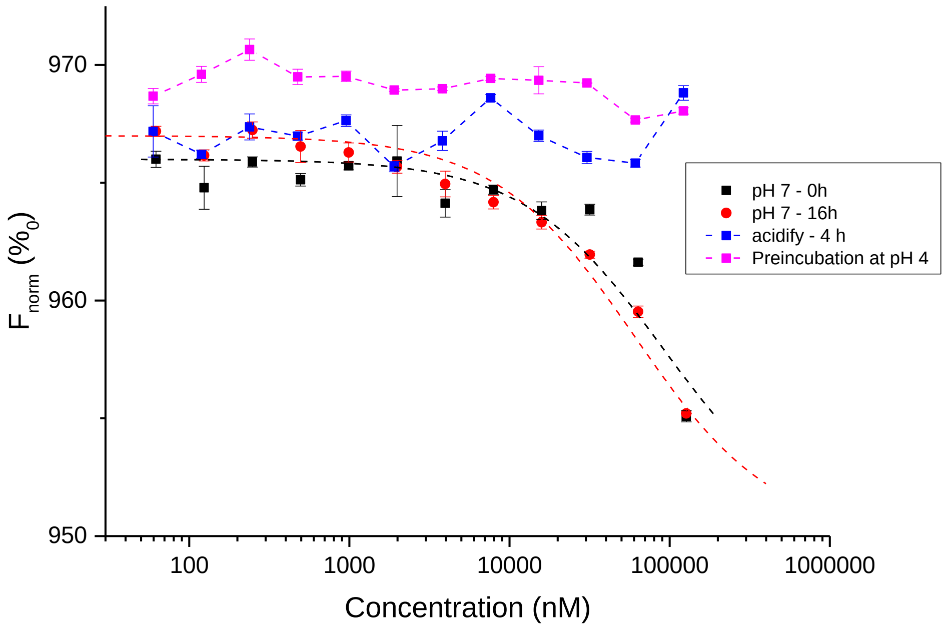

Figure S8. Binding of B-C3 to SST3-Avi determined by microscale thermophoresis (MST). Binding was observed at pH 7 upon immediate incubation of fluorescent labelled B-C3 and SST3-Avi and binding was retained after 16 h, indicating stability of SST3-Avi-C3 (black and red). After acidification, no binding was observed, which suggests the release of the C3 (blue). B-C3 preincubated in pH 4 buffer was applied as a negative control and showed no binding (magenta).

Stability of SST3-Avi-C3 in human serum. 5 µl of SST3-Avi-C3 (32 µM in 25 mM HEPES, pH 7.3) was added to 5 µl of 20% human serum in 25 mM HEPES, pH 7.3 and incubated for 2, 4, 8 and 16 h. As a control, B-C3 (32 µM, ~25 kDa) was incubated in a similar condition for 2 h. Thereafter, 40 µl of HEPES buffer was added to the solutions. An aliquot of 9 µl of protein solution was used and added to 15 µl of 40% glycerol in HEPES buffer for subsequent analysis with SDS-PAGE under without heating. An aliquot of the solution of 20% human serum in HEPES buffer was also applied as control. In addition, SST3-Avi-C3 in buffer solution without human serum was also analyzed with and without heating as described above as control. The gel was then blotted on a nitrocellulose membrane and biotinylated components were detected using streptavidin/peroxidase conjugate to determine the stability of SST3-Avi-C3. Native PAGE was not feasible for quantification due to the positive pI of avidin.[3]

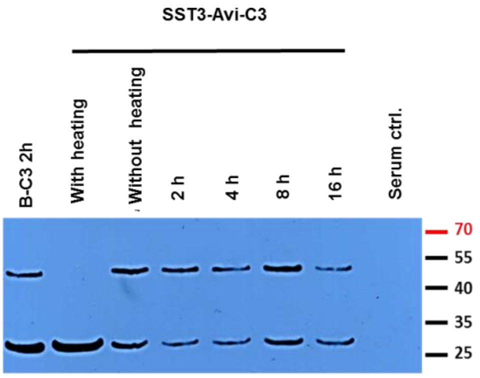

Figure S9. Stability in human serum. Western blot analysis showed that there is no significant increase of free B-C3 up to 16 h of incubation from SST3-Avi-C3. SST3-Avi-C3 in buffer, B-C3 and serum were applied as controls. The lower band at ~ 25 kDa corresponds to B-C3 whereas the upper band at ~50 kDa is presumably due to dimeric C3.

Analysis of the effects mediated by C3 on A549 cells. A549 cells were seeded in 96 well plastic dishes and were incubated with increasing amounts of C3 (200, 400, 600, 1000, 1500 nM) in DMEM medium at 37°C and 5% CO_2. For control, cells were left untreated. After 24 h incubation, phase contrast pictures were taken with a Zeiss Axiovert microscope (20x magnification), representative pictures are shown. The effects on the cells were determined via quantification of degree of change in cells morphology. For quantitative analysis, the percentage of A549 cells showing C3-morphology was determined from 3 independent samples treated in the same way (n = 3, values are given as mean ± SD, average number of cells per well: 130, blinded evaluation of cell morphology).

Detection of cell-associated C3. A549 cells were seeded overnight in a 24 well plate and then incubated with 350 nM SST3-Avi-C3, 350 nM C3, 350 nM B-C3 or were left untreated. After 24 h, cells were washed several times, lysed in hot 2.5 x Lämmli-buffer + DTT and heated at 95 °C for 10 min. Next, lysates were separated by SDS-PAGE, transferred onto a nitrocellulose membrane *via* Western blot and membrane was blocked with 5% dry milk in PBS-T for 30 min. Cell-associated C3 was stained with a rabbit-anti-C3-antibody (1:10000 in PBS-T for 30 min) in combination with a goat-anti-rabbit-antibody (1:2500 in PBS-T for 30 min)) and subsequently visualized by chemiluminescence reaction using Immobilon Western Chemiluminescent HRP Substrate® from Millipore (Schwalbach, Germany) according to the manufacturer's instruction. Equal protein loading was confirmed by actin-staining with a mouse-anti-actin-antibody (1:10000 in PBS-T for 30 min) in combination with a chicken-anti-mouse-HRP-antibody (1:2500 in PBS-T for 30 min) and subsequent chemiluminescence reaction.

Analysis of the effects mediated by SST3-Avi-C3 on A549 cells. For the cytotoxicity experiments, cells were seeded in 96 well plastic dishes and were incubated with SST3-Avi-C3 in DMEM medium at 37°C and 5% CO_2. As control, cells were incubated with SST3-Avi-C3, SST3-Avi or C2IN-C3/C2IIa or were left untreated. After the indicated incubation times, pictures of the cells were acquired. The effects on cell morphology were determined by counting the cells showing the characteristic C3-morphologyin in a blinded manner by two independent investigators. The values were given as mean ± SD (n=3).

***In vitro* ADP-ribosylation of Rho in A549 cells.** SSTR2-positive A549 cells were incubated together with either SST3-Avi-C3, SST3-Avi, C2IN-C3/C2IIa or were left untreated for 24 h, harvested in ADP-ribosylation buffer containing 1 mM DTT, 5 mM $MgCl_2$, 1 mM EDTA, 20 mM Tris-HCl pH 7.5, and lysed using a 25 gauge needle. Equal amounts of protein were incubated with 10 mM biotin-labelled NAD^+ and 300 ng C3 for 30 min at 37°C. The enzyme reaction was stopped by the addition of SDS sample buffer and subsequent heating at 95 °C for 10 min. Followed by SDS-PAGE and Western blot transfer onto a nitrocellulose membrane, the biotin-labeled ADP-ribosylated Rho proteins were detected with peroxidase-coupled streptavidin (1:2500) and subsequent chemiluminescence reaction according to the manufacturer's instruction. Comparable protein loading was confirmed by actin-staining as described above.

Internalization of SST3-Avi-C3 into mammalian cells. For the immunocytochemical analysis A549 cells were seeded in 8-well plates (ibidi GmbH, Munich, Germany) with a density of 30,000 cells per well in 300 µl. The cells were incubated at 37°C and 5% CO_2 overnight in DMEM medium. Then cells were incubated for 24 h with SST3-Avi-C3, SST3-Avi or left untreated as control. Next, cells were washed, fixed with 4% paraformaldehyde, permeabilized using 0.4% Triton X-100 and blocked with 5% dry milk powder in PBS-T. To visualize C3, the toxin was stained with a rabbit-anti-C3-antibody (1:2000 in 5% dry milk in PBS-T for 30 min) in combination with an Alexa Fluor® 647-coupled goat-anti-rabbit secondary antibody (1:2000 in 5% dry milk in PBS-T). The SST3-Avi transporter was FITC labelled. Imaging was then performed using a LCSM.

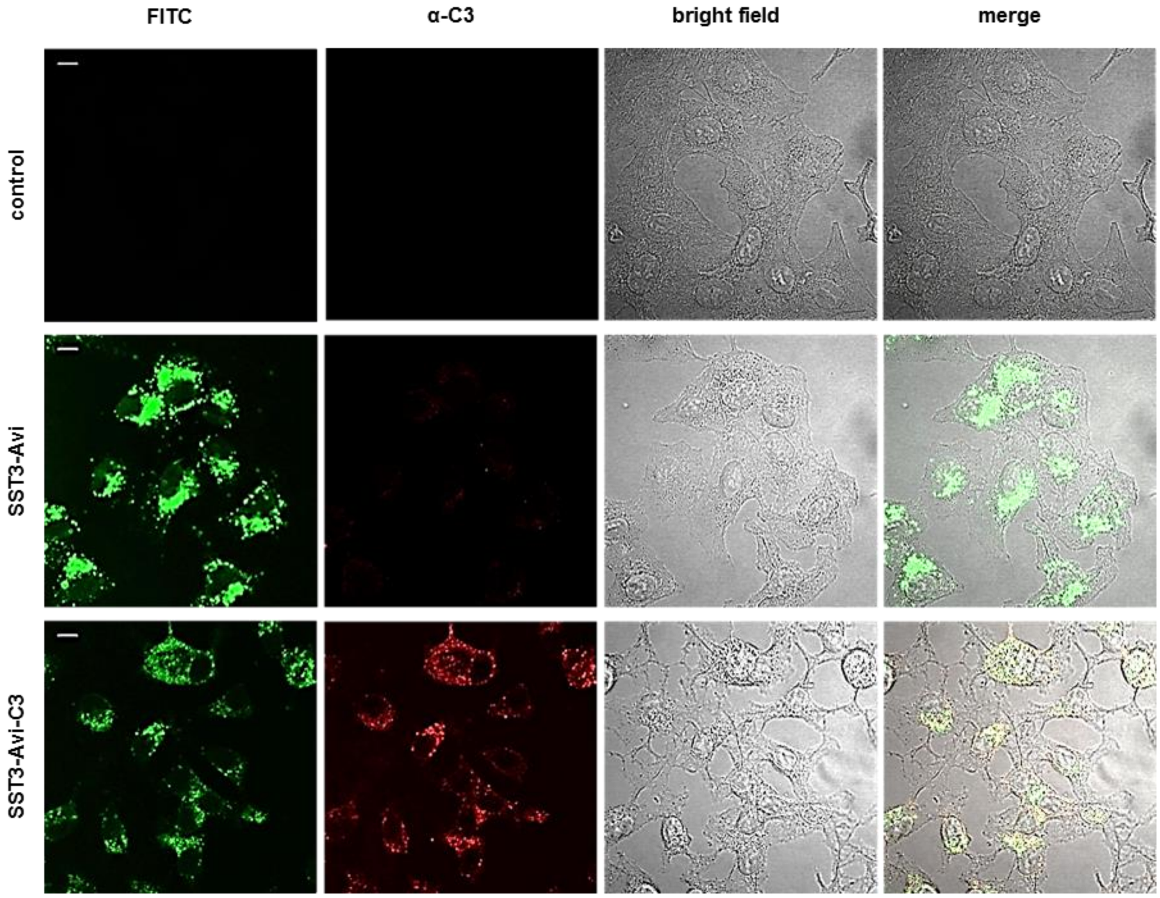

Figure S10. **LSM analysis shows the delivery and internalization of C3 enzyme into A549 cells.** A549 cells grown in eight well µ-Slide-plates (ibiTreat) were incubated at 37 °C with SST3-Avi-C3 (350 nM). As control, cells were treated with SST3-Avi (400 nM) or were left untreated. After 24 h, cells were washed, fixed, permeated and stained with a primary rabbit-antibody against C3 (α-C3) and visualized using an Alexa 647-coupled secondary antibody (red). The transporter was labelled with FITC (green). Cells were then analyzed by fluorescence microscopy. Scale bars correspond to 10 µm.

Chick chorioallantoic membrane (CAM) assay. The CAM xenotransplantation model is a reproducible and efficient model[3,4] conforming to the 3R principle to reduce mammalian experiments. It allows studying various aspects of tumor biology and response to treatment, both by direct monitoring *in ovo* and subsequent immunohistochemical examination of tumor tissue after extraction to quantify specific proteins indicative of treatment-associated effects.[5] The CAM model represents a very suitable system because the capillary network of the CAM is prone to new blood vessel formation, which is essential for the embryonic gas exchange. Therefore, the CAM model has been recommended by the United States Food and Drug Administration (FDA) as preclinical model for *in vivo* studies on angiogenesis.

Chorioallantoic membrane (CAM) *in vivo* xenografts. 1 x 10^6 A549 cells were grafted in medium/Matrigel (1:1, v/v) onto the chorioallantoic membrane (CAM) of chick eggs 8 days after fertilization. One day later, SST3-Avi-C3 (350 nM) or controls/chemotherapeutics (NaCl 0.9%, SST3-Avi (400 nM), B-C3 (350 nM, SI, Fig. S12), bevacizumab (34 µM), doxorubicin (100 µM)) were topically applied in 20 µl. After 24 h, tumor xenografts were collected and embedded in paraffin for immunohistological staining (i.e., HE, hematoxylin and eosin) to reveal morphology; Ki-67 proliferation marker to identify the growth fraction within the tumor xenografts; TUNEL staining, TdT-mediated dUTP-biotin nick end labeling, to tag nuclei of apoptotic cells; desmin staining to identify new blood vessel formation. To calculate the proportion of marker positive and negative cells, 200-790 cells per tumor were evaluated.

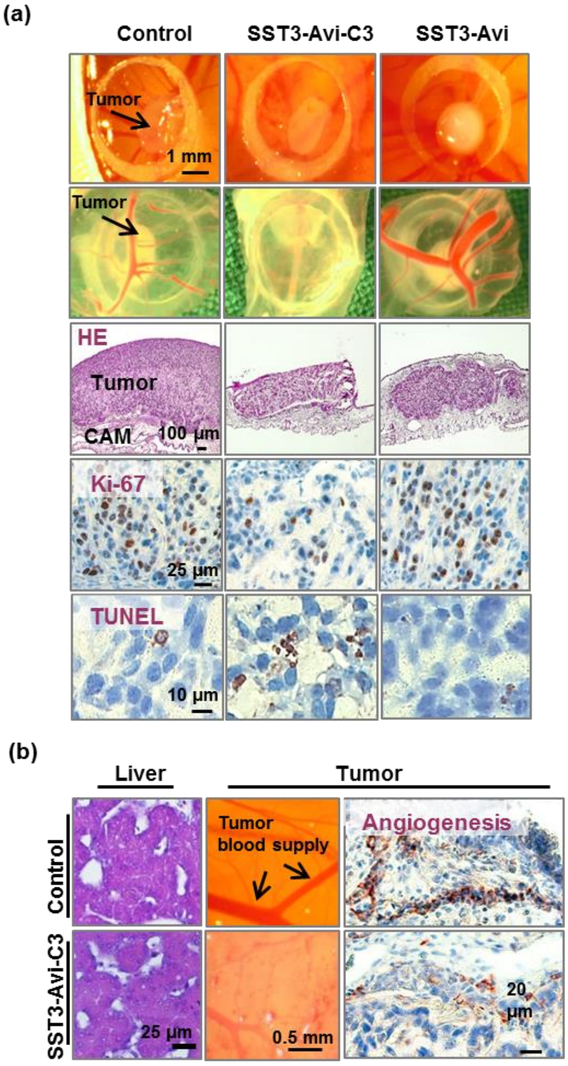

Figure S11. SST3-Avi-C3 selectively inhibits growth of A549 lung cancer xenografts *in vivo*. 1 x 10^6 A549 cells were xenotransplanted on the chorioallantoic membrane of chick eggs 8 days after fertilization. 1 day later, 20 µl of SST3-Avi-C3 (350 nM) and of controls (NaCl

0.9%, 400 nM SST3-Avi) were topically applied. After 24 h, tumor xenografts were collected and histopathologically analyzed; HE, hematoxylin and eosin, original magnification 50x; Ki-67 antigen, red-brown nuclei indicative of proliferating cells, original magnification 200x; angiogenesis marker desmin, original magnification 200x; TUNEL, DNA fragmentation indicated by brown apoptotic bodies, original magnification 400x. For calculation of the proportion of marker positive and negative cells, 200-790 cells per tumor were evaluated. Data are mean ± SEM of 11-13 eggs per group. (**a**) Representative macroscopic pictures of A549 lung cancer xenografts; tumors treated *in ovo* (upper row) and immediately after removal (2nd row); sections of tumor xenografts (3rd row, HE); proliferation marker Ki-67 (4th row) and apoptosis marker TdT (bottom row). (**b**) Representative sections of chick embryo liver sections (HE, 1st column) and macroscopic pictures of tumor nourishing CAM blood vessels 24 h after treatment with SST3-Avi-C3 or control (2nd column) with correlating immunohistochemical staining for the angiogenesis marker desmin (3rd column).

Effects of B-C3 and Cys-C3 on the growth of lung cancer xenografts *in vivo*. 1 x 10^6 A549 cells stably expressing firefly luciferase (A549 Red-FLuc) were xenografted onto the chorioallantoic membrane (CAM) of fertilized chick eggs and topically treated with B-C3 (350 nM), Cys-C3 (350 nM) or control (NaCl 0.9%). Bioluminescence was measured 24 h later, 5 min after addition of D-luciferin 0.75 mg/ml with integration time 1 s, F/Stop 8. Afterwards, tumor xenografts were collected and embedded in paraffin for immunohistological staining.

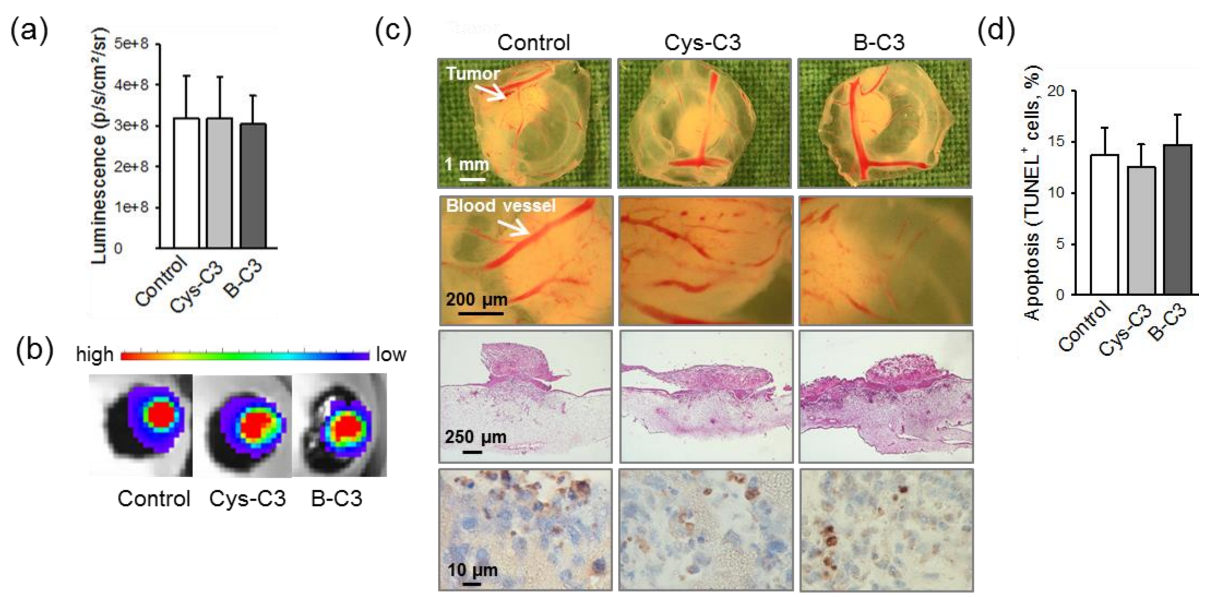

Figure S12. B-C3 and Cys-C3 do not affect the growth of lung cancer xenografts *in vivo*.

(**a**) Tumor growth indicated by expression of luciferase of cancer xenografts was measured by an IVIS *in vivo* Imaging System. The data are mean ± SEM of n=5-7 eggs/group. (**b**) Representative pictures of luciferase expressing tumor xenografts *in ovo*. (**c**) Representative pictures of tumor xenografts after extraction (1st row), tumor microvasculature 72 h after application of Cys-C3 or B-C3 (2nd row). Hematoxilin and eosin, HE stained A549 xenografts (3rd row, original magnification 50x), and apoptotic (TdT positive) cells (4th row, original magnification 400x). (**d**) Apoptotic TdT$^+$ cells within tumor xenografts (4-6 tumors/group). In each tumor, 2 fields of view (176-613 cells) were evaluated.

SST3-Avi-C3 does not affect lung cancer cell viability *in vitro*. 5 x 10^3 Red-FLuc$^+$ A549 cells/well were seeded in 96 well plates the day before the experiment. The next day, medium was replaced by media containing SST3-Avi-C3 or controls at different concentrations. After 24 h of incubation, cell viability was assessed using XTT.

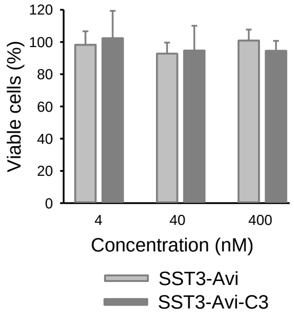

Figure S13. SST3-Avi-C3 does not affect lung cancer cell viability *in vitro*. Cell viability of A549 Red-FLuc cells after 24 h incubation with SST3-Avi-C3 or SST3-Avi (4, 40, 400 nM). Data are mean ± SD of triplicates.

Pretreatment of A549 cells with SST3-Avi-C3 and subsequent administration of doxorubicin.

A549 cells were seeded in 96 well plates with a density of 8500 cells per well in 100 µl. After incubation at 37 °C and 5% CO_2 overnight, cells were pretreated with SST3-Avi-C3 (350 nM), SST3-Avi (400 nM) or DMEM as negative control. After 24 h, cells were washed and then incubated with different concentrations of doxorubicin-HCl (0.1 / 1 / 5 / 20 µM). After another 24 h incubation, cell viability was measured by MTS assay (Cell Titer 96® Aqueous One solution cell proliferation assay, Promega, Mannheim, Germany) according to the manufacturer's instruction (Fig. S14).

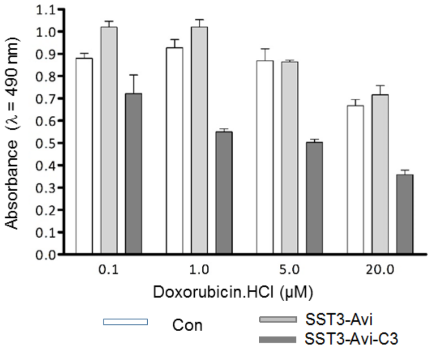

Figure S14. Pre-incubation of cultured A549 cells with SST3-Avi-C3 increases the efficacy of doxorubicin-HCl. A549 cells were pretreated for 24 h with either SST3-Avi (400 nM) or SST3-Avi-C3 (350 nM). For control, cells were left untreated (con). After 24 h, the medium was removed and different concentrations of doxorubicin-HCl were added. The cells were further incubated and after 24 h incubation, the cell viability was measured by MTS assay. Data are given as mean ± SD of triplicates.

Reference

1. Wang, T. *et al.* Cross-conjugation of DNA, proteins and peptides via a pH switch. *Chem. Sci.* **4,** 1889–1894 (2013).
2. Wang, T. *et al.* A disulfide intercalator toolbox for the site-directed modification of polypeptides. *Chem. – A Eur. J.* **21,** 228–238 (2015).
3. Saw, C. L. L., Heng, P. W. S. & Liew, C. V. Chick chorioallantoic membrane as an in situ biological membrane for pharmaceutical formulation development: A review. *Drug Dev. Ind. Pharm.* **34,** 1168–1177 (2008).
4. Ribatti, D. The chick embryo chorioallantoic membrane as a model for tumor biology. *Exp. Cell Res.* **328,** 314–324 (2014).
5. Dohle, D. S. *et al.* Chick ex ovo culture and ex ovo CAM assay: how it really works. *J. Vis. Exp.* 2–7 (2009).
